# Active Fraction Combination From Liuwei Dihuang Decoction Improves Adult Hippocampal Neurogenesis and Neurogenic Microenvironment in Cranially Irradiated Mice

**DOI:** 10.3389/fphar.2021.717719

**Published:** 2021-09-23

**Authors:** Mingxiao Wei, Shufang Feng, Lin Zhang, Chen Wang, Shasha Chu, Tianyao Shi, Wenxia Zhou, Yongxiang Zhang

**Affiliations:** ^1^ School of Life Science and Biopharmaceutics, Shenyang Pharmaceutical University, Shenyang, China; ^2^ State Key Laboratory of Toxicology and Medical Countermeasures, Beijing Institute of Pharmacology and Toxicology, Beijing, China; ^3^ Department of Poisoning and the Treatment, Affiliated Hospital to Academy of Military Medical Sciences (the 307 Hospital), Beijing, China

**Keywords:** traditional Chinese medicine, adult hippocampal neurogenesis, neural stem cells, neurogenic microenvironment, dorsal hippocampus, ventral hippocampus, cranial irradiation, LW-AFC

## Abstract

**Background:** Cranial radiotherapy is clinically used in the treatment of brain tumours; however, the consequent cognitive and emotional dysfunctions seriously impair the life quality of patients. LW-AFC, an active fraction combination extracted from classical traditional Chinese medicine prescription Liuwei Dihuang decoction, can improve cognitive and emotional dysfunctions in many animal models; however, the protective effect of LW-AFC on cranial irradiation–induced cognitive and emotional dysfunctions has not been reported. Recent studies indicate that impairment of adult hippocampal neurogenesis (AHN) and alterations of the neurogenic microenvironment in the hippocampus constitute critical factors in cognitive and emotional dysfunctions following cranial irradiation. Here, our research further investigated the potential protective effects and mechanisms of LW-AFC on cranial irradiation–induced cognitive and emotional dysfunctions in mice.

**Methods:** LW-AFC (1.6 g/kg) was intragastrically administered to mice for 14 days before cranial irradiation (7 Gy γ-ray). AHN was examined by quantifying the number of proliferative neural stem cells and immature neurons in the dorsal and ventral hippocampus. The contextual fear conditioning test, open field test, and tail suspension test were used to assess cognitive and emotional functions in mice. To detect the change of the neurogenic microenvironment, colorimetry and multiplex bead analysis were performed to measure the level of oxidative stress, neurotrophic and growth factors, and inflammation in the hippocampus.

**Results:** LW-AFC exerted beneficial effects on the contextual fear memory, anxiety behaviour, and depression behaviour in irradiated mice. Moreover, LW-AFC increased the number of proliferative neural stem cells and immature neurons in the dorsal hippocampus, displaying a regional specificity of neurogenic response. For the neurogenic microenvironment, LW-AFC significantly increased the contents of superoxide dismutase, glutathione peroxidase, glutathione, and catalase and decreased the content of malondialdehyde in the hippocampus of irradiated mice, accompanied by the increase in brain-derived neurotrophic factor, insulin-like growth factor-1, and interleukin-4 content. Together, LW-AFC improved cognitive and emotional dysfunctions, promoted AHN preferentially in the dorsal hippocampus, and ameliorated disturbance in the neurogenic microenvironment in irradiated mice.

**Conclusion:** LW-AFC ameliorates cranial irradiation–induced cognitive and emotional dysfunctions, and the underlying mechanisms are mediated by promoting AHN in the dorsal hippocampus and improving the neurogenic microenvironment. LW-AFC might be a promising therapeutic agent to treat cognitive and emotional dysfunctions in patients receiving cranial radiotherapy.

## Introduction

People are subjected to irradiation exposure commonly during the process of radiodiagnosis and radiotherapy (Brenner and Hall, 2007; Manda and Reiter, 2010; Hladik and Tapio, 2016). A typical example is cranial irradiation which is essential for the treatment of lots of cancer types, such as primary and metastatic brain tumours, and many head and neck malignancies (Monje, 2008). Although cranial irradiation is effective in cancer therapy, it can produce cognitive and emotional dysfunctions which seriously impair the life quality of patients (Son et al., 2015). There are currently no effective clinical interventions for these cognitive and emotional dysfunctions (D’Antonio et al., 2014); therefore, finding therapeutic drugs to ameliorate these cognitive and emotional dysfunctions has become very important.

Cognitive and emotional dysfunctions in patients after treatment with cranial irradiation point to hippocampal damage, and recent evidence indicates that the impairment of adult hippocampal neurogenesis (AHN) is one of the most important mechanisms involved in cranial irradiation–induced cognitive and emotional dysfunctions (Raber et al., 2004; Limoli et al., 2007; Naylor et al., 2008; Acharya et al., 2011). It is accepted that the hippocampus is functionally segregated into the dorsal region mainly implicated in cognitive function and the ventral region crucial for emotional process. In the mammalian brain, AHN occurs in the dentate gyrus of the hippocampus along the dorsal–ventral axis that continuously results in the generation of newborn neurons. AHN is a unique form of structural and functional plasticity and plays an important role in both cognition and emotion (Ming and Song, 2005; Imayoshi et al., 2008; Balu and Lucki, 2009). However, AHN can be severely impaired by cranial irradiation even at low doses, indicating particular vulnerability (Tada et al., 2000; Monje et al., 2002; Mizumatsu et al., 2003). It is also reported that the neurogenic microenvironment (neurogenic niche) in the hippocampus modulates the different processes of neural stem cell development and cranial irradiation damages the neurogenic microenvironment, which mainly correlates with the reduced AHN (Monje and Palmer, 2003; Fike et al., 2007; Ming and Song, 2011; Huang T.-T. et al., 2012). The extreme radiosensitivity of neural stem cells and alterations in the neurogenic microenvironment constitute critical factors in the cognitive and emotional dysfunctions following cranial irradiation. It seems probable that successful interventions for these cognitive and emotional dysfunctions will involve both protection of AHN and drug-based regulation of the neurogenic microenvironment (Monje et al., 2002; Jenrow et al., 2011).

Many traditional Chinese medicine (TCM) prescriptions “nourishing” in the TCM theory system have the effects of improving cognition and emotion (Nishiyama et al., 1994; Itoh et al., 1998; Lin et al., 2003; Yang et al., 2006; Zhang et al., 2016; Hu et al., 2020; Shang et al., 2020). Furthermore, TCM shows good prospects in promoting AHN (Zhang et al., 2014; Sreenivasmurthy et al., 2017; Chen et al., 2019). Liuwei Dihuang decoction (LW), a classical TCM prescription, has been used for the treatment of various diseases with features of “kidney yin” deficiency for about 900 years in China (Huang Y. et al., 2012; Chen et al., 2021). LW consists of six traditional Chinese herbs, *Rehmannia glutinosa* (Gaertn.) DC. [Orobanchaceae; Rehmanniae radix], *Dioscorea oppositifolia* L. [Dioscoreaceae; Dioscoreae rhizoma], *Cornus officinalis* Siebold and Zucc. [Cornaceae; Corni fructus], *Alisma plantago-aquatica* L. [Alismataceae; *Alismatis* rhizoma], *Wolfiporia extensa* (Peck) Ginns (syn. *Poria cocos* (Schw.)).Wolf [Polyporaceae; Poria], and *Paeonia* × *suffruticosa* Andrews [Paeoniaceae; Moutan cortex], mixed in a ratio of 8:4:4:3:3:3 (Zhou W. et al., 2016). LW is included in the Chinese Pharmacopoeia (2010 version). In the clinic, LW has been used to prevent and treat cognitive dysfunction, depression, climacteric syndrome, cancer, diabetes, and cardiovascular disease (Cheng et al., 2019). A previous study reported that LW could promote AHN in adult rats (Lee et al., 2005). LW-AFC is a new formula of the main active fraction combination extracted from LW. There are three active components in LW-AFC, including polysaccharide fraction (LWB-B), glycoside fraction (LWD-B), and oligosaccharide fraction (CA-30) (Wang et al., 2017a). Previous studies showed that LW-AFC facilitated learning and memory, reduced the amyloid-β (Aβ) plaque load, and restored the imbalance of the hypothalamic–pituitary–adrenal (HPA) axis in the mice model of Alzheimer’s disease (Wang et al., 2016; Wang et al., 2017b). LW-AFC also ameliorated long-term potentiation (LTP) impairment in mice induced by acute stress or lipopolysaccharide (Zeng et al., 2019b; Huang et al., 2019). Moreover, LW-AFC was found to enhance learning and memory performance and reduce anxiety- and depression-like behaviour in mice induced by chronic stress (Shen et al., 2018; Zhu et al., 2018; Zeng et al., 2019a). It showed that LW-AFC improved cognitive and emotional dysfunctions in many animal models; however, whether LW-AFC possesses therapeutic effects on cognitive and emotional dysfunctions induced by cranial irradiation has not been reported. Meanwhile, it is not clear whether LW-AFC has beneficial effects on cranial irradiation–induced impairments in AHN along the dorsal–ventral axis and in the neurogenic microenvironment.

The present study aimed at investigating the protective effects of LW-AFC on cognitive and emotional dysfunctions in cranially irradiated mice and whether the underlying mechanisms were mediated by promoting AHN along the dorsal–ventral axis of the hippocampus and ameliorating the neurogenic microenvironment. Given that the dorsal hippocampus is mainly implicated in cognitive function and the ventral hippocampus is predominantly involved in emotional regulation, AHN is not regulated uniformly along the dorsal–ventral axis depending on the stimulus presented (Felice et al., 2012; O'Leary et al., 2012; Vivar et al., 2016). Therefore, in this study, we detected the effect of LW-AFC on AHN in both the dorsal and ventral hippocampus of cranially irradiated mice. Based on the view of regulating AHN and the neurogenic microenvironment, this study should offer a potentially effective treatment for cranial irradiation–induced cognitive and emotional dysfunctions from the resource of TCM and provide scientific evidence for facilitating its application in the clinic. In addition, this study developed a scientific method to investigate whether TCM and its bioactive components could promote AHN accurately in different subregions of the hippocampus along the dorsal–ventral axis.

## Materials and Methods

### Animals

Male C_57_BL/6J mice (20–22 g, 8 weeks old) were acquired from Beijing Sibeifu Animal Company (Animal Licence No. SCXK 2016-0002; Beijing, China). The mice were housed in groups (4 per cage) and kept under the standard condition (12/12 h light/dark cycle, 22 ± 1°C, food and water ad libitum). All mice adapted to ambient rearing conditions for 7 days before the experiments. All efforts were made to ensure the respect and comfort of the animals. All of the animal's care and treatment, feeding management, and experimental protocols were approved by Institute Animal Care and Use Committee (IACUC) of the National Beijing Center for Drug Safety Evaluation and Research (NBCDSER) (No. 2018-030), in compliance with the National Institutes of Health (NIH) Guide for the Care and Use of Laboratory Animals (NIH Publications No. 80-23, revised 1996).

### Drugs and Reagents

Bromodeoxyuridine (BrdU) (B5002), Triton X-100 (T8787), and bovine serum albumin (BSA) (A3858) were purchased from Sigma-Aldrich (St. Louis, MO, United States). Paraformaldehyde (CAS: 30525-89-4), sucrose (CAS: 57-50-1), hydrochloric acid (HCl) (CAS: 7647-01-0), boric acid (CAS: 10043-35-3), and alcohol (CAS: 64-17-5) were obtained from Sinopharm Chemical Reagent Co., Ltd. (Shanghai, China). Sodium pentobarbital (Lot. 34-06-12) was purchased from Shanghai Chemical Reagent Assembly Factory (Shanghai, China). These drugs and reagents were all of analytically pure grade.

Anti-BrdU antibody (ab6326) was obtained from Abcam (Cambridge, United Kingdom), and anti-doublecortin (DCX) antibody (#4604) was purchased from Cell Signaling Technology (Boston, MA, United States). Alexa Fluor 488-conjugated donkey anti-rat IgG (A21208) and Alexa Fluor 594-conjugated goat anti-rabbit IgG (A11037) were acquired from Thermo Fisher Scientific (Waltham, MA, United States). The fluorescent mounting medium containing DAPI (ZLI-9557) was purchased from Zhongshan Jinqiao Biotechnology Co., Ltd. (Beijing, China). Phosphate-buffered saline (PBS) (AR0030) was obtained from Boster Biological Technology Co., Ltd. (Wuhan, China). Normal saline (Lot. 1811242004) was purchased from Shijiazhuang Siyao Co., Ltd. (Shijiazhuang, China).

### The Preparation of LW-AFC

The original herbs of Liuwei Dihuang decoction (LW) were purchased from Beijing Tongrentang Pharmacy (Beijing, China), which included 32% *Rehmannia glutinosa* (Gaertn.) DC. [Orobanchaceae; Rehmanniae radix] (Lot. 20150130), 16% *Dioscorea oppositifolia* L. [Dioscoreaceae; Dioscoreae rhizoma] (Lot. 1503028), 16% *Cornus officinalis* Siebold and Zucc. [Cornaceae; Corni fructus] (Lot. 20151126), 12% *Alisma plantago-aquatica* L. [Alismataceae; Alismatis rhizoma] (Lot. 20160116), 12% *Wolfiporia extensa* (Peck) Ginns (syn. *Poria cocos* (Schw.)). Wolf [Polyporaceae; Poria] (Lot. 1601001), and 12% *Paeonia* × *suffruticosa* Andrews [Paeoniaceae; Moutan cortex] (Lot. 20160526) at a proportion of 8:4:4:3:3:3. The total dry weight was 3 kg. They were authenticated by Professor Yimin Zhao and Shanyi Qiao (Department of Phytochemistry, Beijing Institute of Pharmacology and Toxicology) according to Chinese Pharmacopoeia (2010 version). The voucher specimens were stored at the Department of Phytochemistry, Beijing Institute of Pharmacology and Toxicology.

LW-AFC was prepared from LW. The details are displayed in Supplementary Materials 2. We briefly describe the method of preparation here. Six herbs of LW, including *Rehmannia glutinosa* (Gaertn.) DC. [Orobanchaceae; Rehmanniae radix], *Dioscorea oppositifolia* L. [Dioscoreaceae; Dioscoreae rhizoma], *Cornus officinalis* Siebold & Zucc. [Cornaceae; Corni fructus], *Alisma plantago-aquatica* L. [Alismataceae; Alismatis rhizoma], *Wolfiporia extensa* (Peck) Ginns (syn. *Poria cocos* (Schw.)). Wolf [Polyporaceae; Poria], and *Paeonia* × *suffruticosa* Andrews [Paeoniaceae; Moutan cortex], were mixed according to the dry weight ratio of 8:4:4:3:3:3. The mixture of herb materials was decocted with 10 volumes of deionized water with boiling refluxing thrice, 2 h each time. After finishing the extraction, the materials were filtered through a 6-layer gauze to yield three extraction solutions at 50°C, allowed to cool to room temperature, and centrifuged (2500 rpm/min, 25 min). The supernatant filtered from LW was concentrated into a quintessence. The quintessence was then extracted using ethanol to produce the supernatant (LWD), and the sediment left in the deionized water was concentrated into the dried LWB-B. LWD was concentrated, and ethanol was removed. LWD was then dissolved in deionized water and eluted in turn with deionized water and 30% ethanol on microporous adsorptive resins. The 30% ethanol elution of LWD was cryodesiccated into LWD-B, and the water elution of LWD was concentrated and eluted in turn with 5% ethanol and 30% ethanol on an active carbon absorption column. The 30% ethanol elution was then concentrated, ethanol was removed, and the elution was cryodesiccated into the CA-30.

### The HPLC Fingerprint for Quality Control of LW-AFC

LW-AFC includes 20.3% LWB-B, 15.1% LWD-B, and 64.6% CA-30 at a dry weight ratio. LWD-B mainly contains loganin, loganic acid, morroniside, sweroside, paeoniflorin, acteoside, isoacteoside, jionoside A_1_, jionoside A_2_, jionoside B_1_, jionoside B_2_, and 5-hydroxymethyl-furaldehyde. CA-30 mainly contains mannotriose and stachyose. LWB-B is mainly composed of polygalacturonic acid, rhamnogalacturonic acid polysaccharide, arabinogalactan, and dextran (Cheng et al., 2019).

**FIGURE 1:**
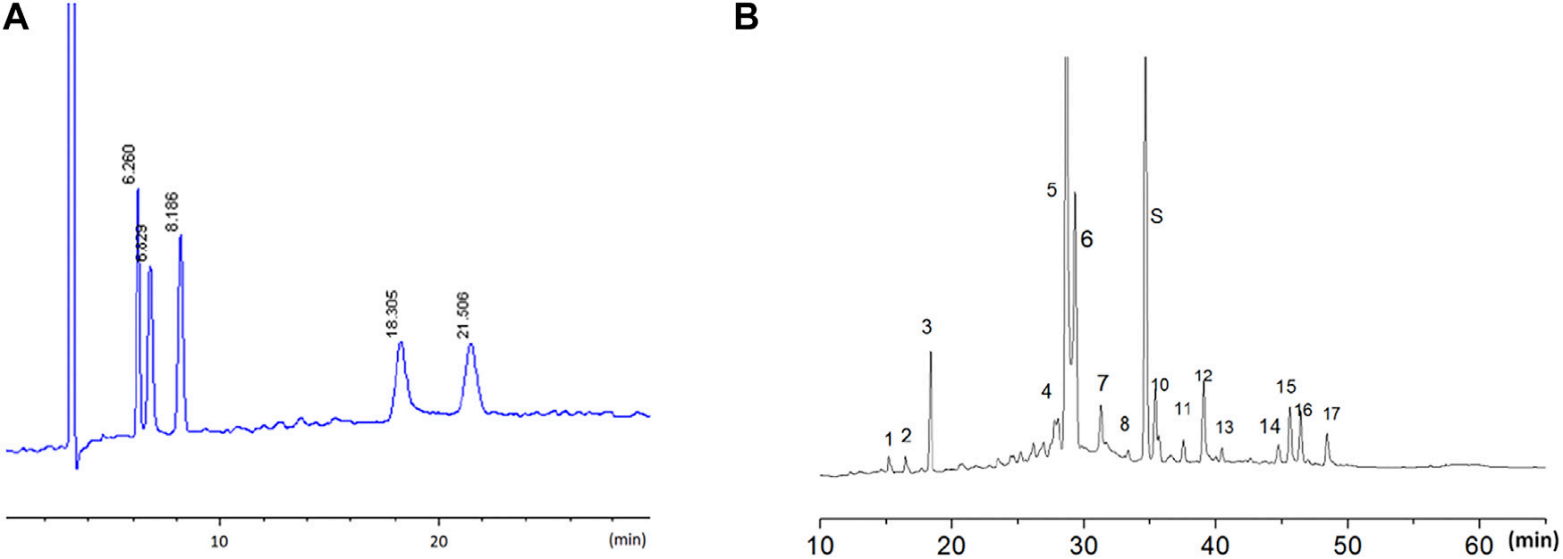
The HPLC fingerprint of LW-AFC. LW-AFC consists of polysaccharide fraction (LWB-B), glycoside fraction (LWD-B), and CA-30. **(A)** The HPLC fingerprint of LWB-B and CA30 mixture. The peak at 6.26, 6.83, 8.19, 18.30, and 21.50 min represents, respectively, fructose, glucose, sucrose, mannotriose, and stachyose. **(B)** The HPLC fingerprint of LWD-B. The peak S represents loganin.

The LW-AFC components were detected using the high-performance liquid chromatography (HPLC) method for quality control. The HPLC fingerprint of LW-AFC is shown in Figure 1. In brief, for LWB-B and CA30 mixture, the chromatographic separation was obtained on a NucleosilNH2 100 Å column; five chromatogram peaks were observed, representing fructose, glucose, sucrose, mannotriose, and stachyose. The retention time of these peaks was 6.260, 6.829, 8.186, 18.305, and 21.506 min, respectively (Figure 1A). For LWD-B, chromatographic separation was obtained on a Diamond C18 column; there were 17 chromatogram peaks in the fingerprint of LWD-B. The S peak represented loganin (Figure 1B).

### Experiment Design

**FIGURE 2:**
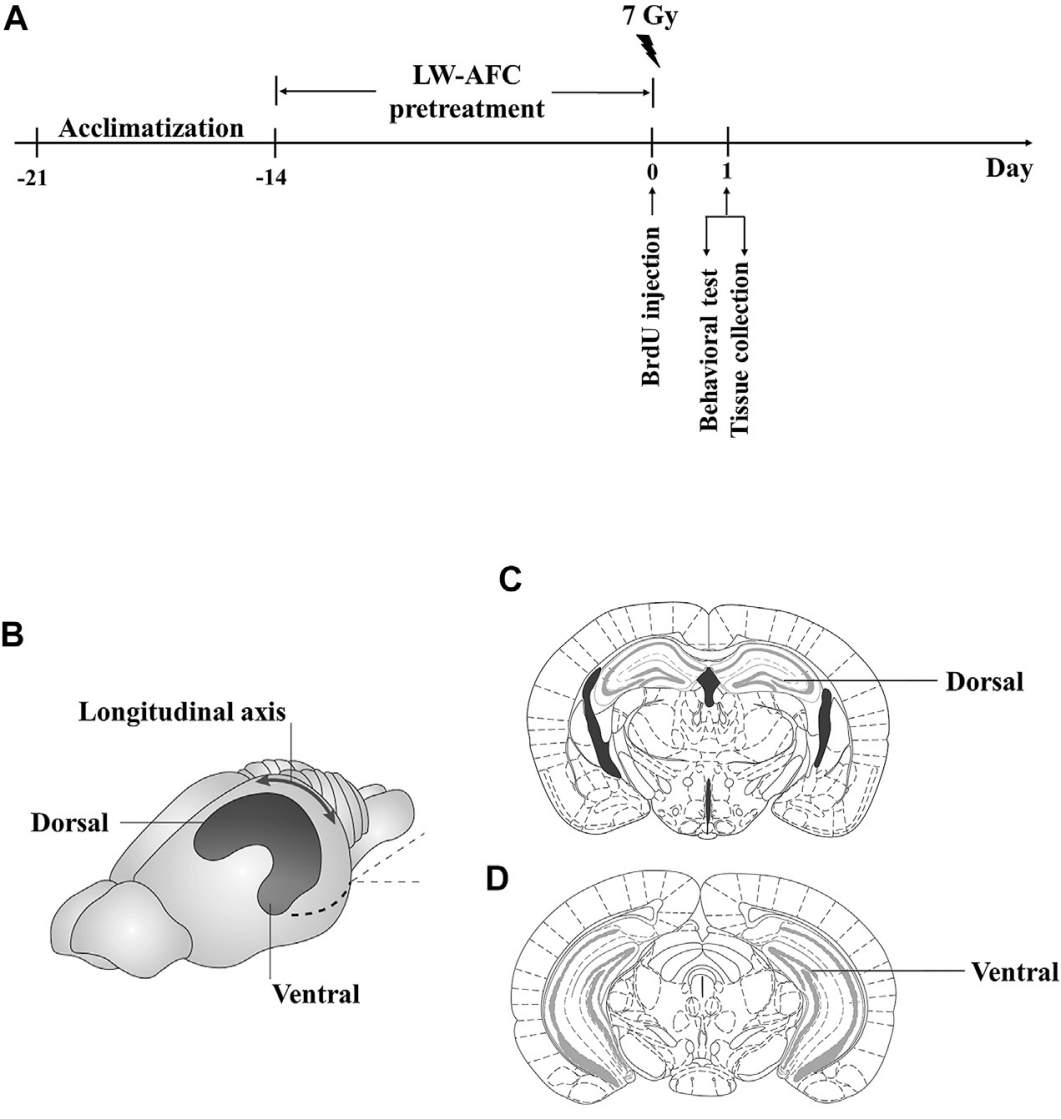
Scheme of the experimental procedure and schematic representation of the dorsal and ventral hippocampus in mice. **(A)** Scheme of the experimental procedure. **(B)** The image of the dorsal and ventral hippocampus along the longitudinal axis. **(C)** The coronal brain section of the dorsal hippocampus. **(D)** The coronal brain section of the ventral hippocampus.

Male C_57_BL/6J mice of 8 weeks old were randomly divided into three groups: control group, irradiation group, and irradiation + LW-AFC group (IR + LW-AFC). Each group had 24 mice. Before irradiation, the mice of the IR + LW-AFC group were given an intragastric administration of LW-AFC (1.6 g/kg body weight) for 2 weeks, while the mice in the control and IR group were given the same volume of distilled water. Next, the mice in the IR and IR + LW-AFC group received 7 Gy γ-ray cranial irradiation, while the mice of the control group were placed into the irradiation device without irradiation. Two days before irradiation, the contextual fear conditioning test was performed, until the end of the experiment. On the first day after irradiation, the open field test and tail suspension test were performed. When the behavioural tests were completed, half of the mice in each group were transcardially perfused and the brains of mice were removed for immunofluorescent staining. In addition, the other half of mice in each group were sacrificed for the multiplex bead analysis and assay of oxidative stress level (Figure 2A).

LW-AFC is a new formula of the main active fraction combination extracted from LW. From the previous experiments in our laboratory, we have demonstrated that LW-AFC (1.6 g/kg) is the optimal resource in improving cognitive and emotional dysfunctions in many animal models (Wang et al., 2016; Wang et al., 2017b; Shen et al., 2018; Zhu et al., 2018; Zeng et al., 2019b; Huang et al., 2019). Therefore, we selected the dose of LW-AFC as 1.6 g/kg in this study.

### Preparation of the Mice Model of Cranial Irradiation

For cranial irradiation, mice were anaesthetised by intraperitoneal injection of 1% sodium pentobarbital in normal saline (0.05 ml/10 g body weight), covered with a sliding shield which protected the body of mice and exposed only the hippocampus to irradiation, and finally irradiated with γ-ray at a single dose of 7 Gy. After the irradiation was completed, the mice were placed into a box containing cotton around the electric heater for rising the temperature quickly so that they wake up as soon as possible. The mice were returned to the home cage for further rearing.

### Bromodeoxyuridine Treatment

After water bath at 37°C for 30 min, BrdU was dissolved in normal saline at a concentration of 2 mg/ml. To test the cell proliferation, mice of all groups received intraperitoneal injection of BrdU solution (50 mg/kg body weight, twice a day, 6 h interval) after irradiation exposure and were perfused for 16 h after the last BrdU injection.

### Preparation of Brain Sections in the Dorsal and Ventral Hippocampus

After perfusion, mice brains were fixed in 4% paraformaldehyde in PBS (0.1 M, pH 7.4) at 4°C for 12 h and dehydrated with 30% sucrose in PBS for 3 days. 40 um-thick sections were cut with a freezing microtome (CM 1950, Leica, Germany) and stored in PBS at 4°C before use. The sections from the dorsal and ventral hippocampus were used for immunofluorescent staining. The dorsal hippocampus and the ventral hippocampus were dissociated according to coordinates: −0.94 to −2.30 mm relative to the bregma for the dorsal hippocampus and −2.46 to −3.80 mm for the ventral hippocampus (O’Leary et al., 2012; Zheng et al., 2017). Five sections were chosen for dorsal hippocampus or ventral hippocampus, respectively.

### Immunofluorescent Staining

The proliferation of neural stem cells and the level of immature neurons are the sensitive indicators for detecting AHN, which can be evaluated by BrdU-positive cells and DCX-positive cells with immunofluorescent staining. For BrdU-positive cell detection, the sections were washed three times in PBS first. Then, the sections were treated with 2M HCl for 1 h at room temperature to denature DNA followed by immersion in 0.1 M boric acid (PH 8.5) for 10 min at room temperature, rinsed three times in PBS, blocked in 0.1% Triton X-100 and 3% BSA in PBS for 1 h, and incubated with rat anti-BrdU antibody (1:200) in PBS with 0.1%Triton X-100 and 3% BSA overnight at 4°C. The next day, after washing in PBS, sections were incubated with Alexa Fluor 488-conjugated donkey anti-rat IgG (1:1,000) in PBS with 0.1%Triton X-100 and 3% BSA for 1 h at room temperature. After washing in PBS again, the sections were mounted on slides with the fluorescent mounting medium containing DAPI, covered with coverslips, and stored at −20°C for further examination.

For DCX-positive cell detection, the sections were washed three times in PBS first. Next, the sections were treated with 0.5%Triton X-100 for 30 min at room temperature, blocked in 0.1% Triton X-100 and 3% BSA in PBS for 1 h, and incubated with rabbit anti-DCX antibody (1:400) in PBS with 0.1%Triton X-100 and 3% BSA overnight at 4°C. The next day, after washing in PBS, sections were incubated with Alexa Fluor 594-conjugated goat anti-rabbit IgG (1:1,000) in PBS with 0.1%Triton X-100 and 3% BSA for 1 h at room temperature. After washing in PBS again, the sections were mounted on slides with the fluorescent mounting medium containing DAPI, covered with coverslips, and stored at −20°C for further examination. BrdU-positive cells and DCX-positive cells were visualized and counted using a Zeiss 880 confocal microscope (Zeiss, Oberkochen, Germany).

### Measurement of Inflammatory, Neurotrophic, and Growth Factors

The proinflammatory factors to be detected include interleukin-1β (IL-1β), IL-6, IL-17A, and tumour necrosis factor alpha (TNF-α). Anti-inflammatory factors include IL-4, IL-10, granulocyte colony–stimulating factor (G-CSF). Neurotrophic and growth factors include brain-derived neurotrophic factor (BDNF), insulin-like growth factor-1 (IGF-1), and vascular endothelial growth factor (VEGF). All of them were detected by multiplex bead analysis as previously described (Wang et al., 2016). The samples of the hippocampus were analysed using Luminex 200™ (Luminex, TX, United States). The levels of these factors were measured using a multifactor detection kit (LXSAMSM-11, R&D Systems, United States) according to the manufacturer’s instructions.

### Assay of the Oxidative Stress Level

The indexes of oxidative stress include glutathione (GSH), glutathione peroxidase (GSH-Px), catalase (CAT), superoxide dismutase (SOD), and malondialdehyde (MDA). These indexes were detected by colorimetry. After hippocampus tissues were homogenized and centrifuged, the supernatant was collected and measured using specific commercial kits related to these indexes (A006-1-1, A005-1-2, A007-1-1, A001-1-1, and A003-1-1; Nanjing Jiancheng Institute of Biological Engineering, China) according to the manufacturer’s instructions.

### Open Field Test

The open field box is made of black polyvinyl chloride (420 × 420 × 420 mm), and there is a center zone in the middle of the box with a permanent marker (205 × 205 mm). The mice were handled every day to be familiar with the experimenter before all behavioural tests. Before the open field test, the mice adapted to the environment of the experimental room for 1 h first. Next, the mice were put into the box of open field gently and explored freely for 5 min. The trajectories of mice were recorded by ANY-maze software (Stoelting Co., United States). When the mice were taken out, the box was wiped with 75% alcohol to remove the odour before the next mice to be tested. ANY-maze software recorded the time of mice in the center zone of the open field within 5 min.

### Contextual Fear Conditioning Test

The contextual fear conditioning test was conducted in chambers with internal dimensions of 30 cm width × 30 cm length × 60 cm height. A house light above the chamber provided illumination. The mice were placed in the environment of the experimental room to adapt for 1 h before the test. The experiment lasted 3 days. The first day was the adaptation period, and the mice were gently placed in the experiment chamber to explore freely for 30 min. The second day was the learning period, and the mice were shocked to absorb the harmful stimulus in the chamber for 5 min. The current parameters were 0.8 mA and 5 times/300 s, the interval time for shock was 60 s, and the duration time for shock was 2 s. The third day was the test period. All the contexts in the experiment chamber remained unchanged. The mice were gently put into the experiment chamber, and the freezing time of mice was recorded within 5 min.

### Tail Suspension Test

The mice accommodated the environment of the experimental room for 1 h before the test. First, the tails of mice were stuck to one side of the adhesive plaster, and the other side of the adhesive plaster was stuck to the hooks of the experiment box as soon as possible. When all mice were suspended to the hangers, the ANY-maze software recorded the trace of mice for 6 min. The immobility time of mice in the latter 4 min in the experiment box was needed for statistical analysis.

### Statistical Analysis

Data were expressed as mean ± standard deviation (S.D.). Data were analysed using the unpaired Student’s *t* test and one-way analysis of variance (ANOVA) with the appropriate post hoc test for multiple comparisons where appropriate. The value of *p <* 0.05 was considered as statistically significant.

## Results

### LW-AFC Pretreatment Improved Cognitive and Emotional Dysfunctions in Cranially Irradiated Mice

To observe the effects of LW-AFC pretreatment for 2 weeks on cognitive and emotional dysfunctions induced by cranial irradiation, the contextual fear conditioning, open field, and tail suspension tests were used on the first day after irradiation.

**FIGURE 3:**
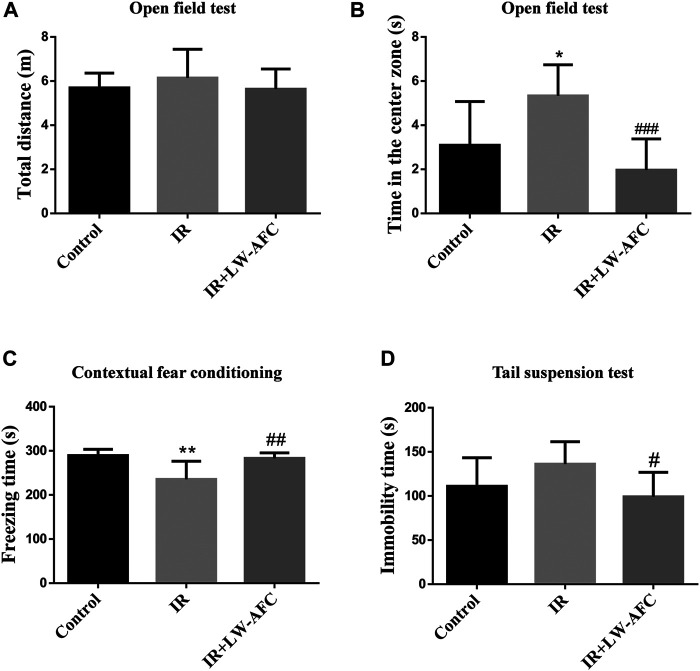
LW-AFC improves cognitive and emotional dysfunctions in IR mice on day 1 after irradiation. **(A)** Quantification of the total distances travelled by mice during the open field test. **(B)** Quantification of the center time of mice during the open field test. **(C)** Quantification of the freezing time of mice during the contextual fear conditioning test. **(D)** Quantification of the immobility time of mice during the tail suspension test. The values denote mean ± S.D., *n* = 8. *p < 0.05 and **p < 0.01, versus the control group; ^#^ *p <* 0.05, ^##^ *p <* 0.01, and ^###^ *p <* 0.001, versus the IR group. Abbreviation: IR, irradiation.

In the open field test, there was no difference in the total distances travelled by mice among the three groups, suggesting that the locomotor activity of mice was not changed (Figure 3A). A significant increase in the time in the center zone was observed in IR mice compared with the control mice, and this change was restored by LW-AFC pretreatment (*p* < 0.001) (Figure 3B). The results revealed that cranial irradiation reduced the anxiety level in mice and LW-AFC reversed cranial irradiation–induced reduction in the anxiety level.

In the contextual fear conditioning test, compared with the control mice, the freezing time of IR mice was significantly reduced and this change could be reversed by LW-AFC pretreatment (*p* < 0.01) (Figure 3C). The results demonstrated that cranial irradiation impaired the contextual fear memory in mice and LW-AFC significantly improved cranial irradiation–induced impairment in the contextual fear memory.

In the tail suspension test, compared with the control mice, the immobility time in IR mice displayed an increasing tendency. The immobility time in IR mice pretreated with LW-AFC was significantly decreased compared with the IR mice (*p* < 0.05) (Figure 3D). The results suggested that cranial irradiation tended to induce depression-like behaviour in mice and LW-AFC reduced depression-like behaviour in IR mice.

Collectively, cranial irradiation led to cognitive and emotional dysfunctions in mice on the first day after irradiation, and these dysfunctions could be ameliorated significantly by LW-AFC.

### LW-AFC Pretreatment Increased the Number of Proliferative Neural Stem Cells in the Dorsal Hippocampus of Irradiated Mice

**FIGURE 4:**
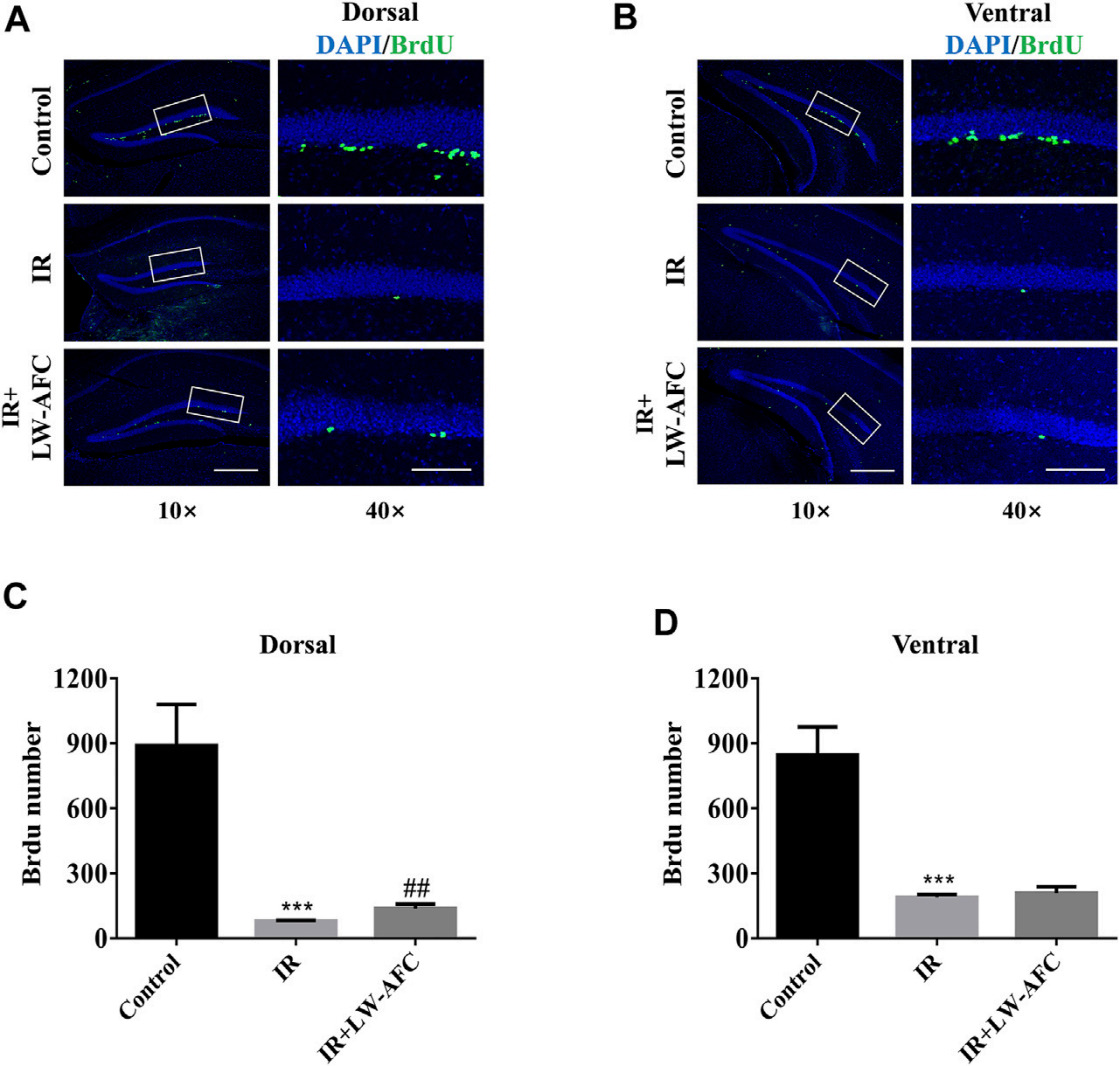
LW-AFC promotes the proliferation of neural stem cells preferentially in the dorsal hippocampus of IR mice on day 1 after irradiation. Representative immunofluorescence images of BrdU-positive cells in the **(A)** dorsal hippocampus and the **(B)** ventral hippocampus. The area of white square frame in the left image indicates the enlarged image of BrdU-positive cells in the right. The confocal images in the left are of 10×, and the enlarged images in the right are of 40×. Quantification of BrdU-positive cells in the **(C)** dorsal hippocampus and the **(D)** ventral hippocampus. The scale bar in the left image = 300 μm, and the scale bar of the enlarged image = 100 μm. The values denote mean ± S.D., *n* = 4. ***p < 0.001, versus the control group; ^##^ *p <* 0.01, versus the IR group. Abbreviation: IR, irradiation.

To assess the effect of LW-AFC pretreatment on the proliferation of neural stem cells in the dentate gyrus of the hippocampus along the dorsal–ventral axis in IR mice, the number of BrdU-positive cells visualized by immunofluorescence staining was quantified. BrdU is a thymine nucleoside analogue, which can replace thymine (T) to penetrate into the replicating DNA molecules during cell proliferation (Martel et al., 2016). A schematic representation of the dorsal and ventral hippocampus is exhibited in Figures 2B–D. On the first day after irradiation, compared with the control mice, the number of BrdU-positive cells in the dorsal hippocampus of IR mice was significantly decreased and this change could be improved significantly by LW-AFC pretreatment (*p* < 0.01) (Figures 4A,C). Compared with the control mice, a significant decrease in the number of BrdU-positive cells in the ventral hippocampus was observed in IR mice; however, the LW-AFC pretreatment did not improve this change (Figures 4B,D). The results showed that the proliferation of neural stem cells in the hippocampus along the dorsal–ventral axis was significantly damaged on the first day after irradiation and LW-AFC significantly improved cranial irradiation–induced impairment in the proliferation of neural stem cells in the dorsal hippocampus with no effect in the ventral hippocampus.

### LW-AFC Pretreatment Increased the Number of Immature Neurons in the Dorsal Hippocampus of Irradiated Mice

**FIGURE 5:**
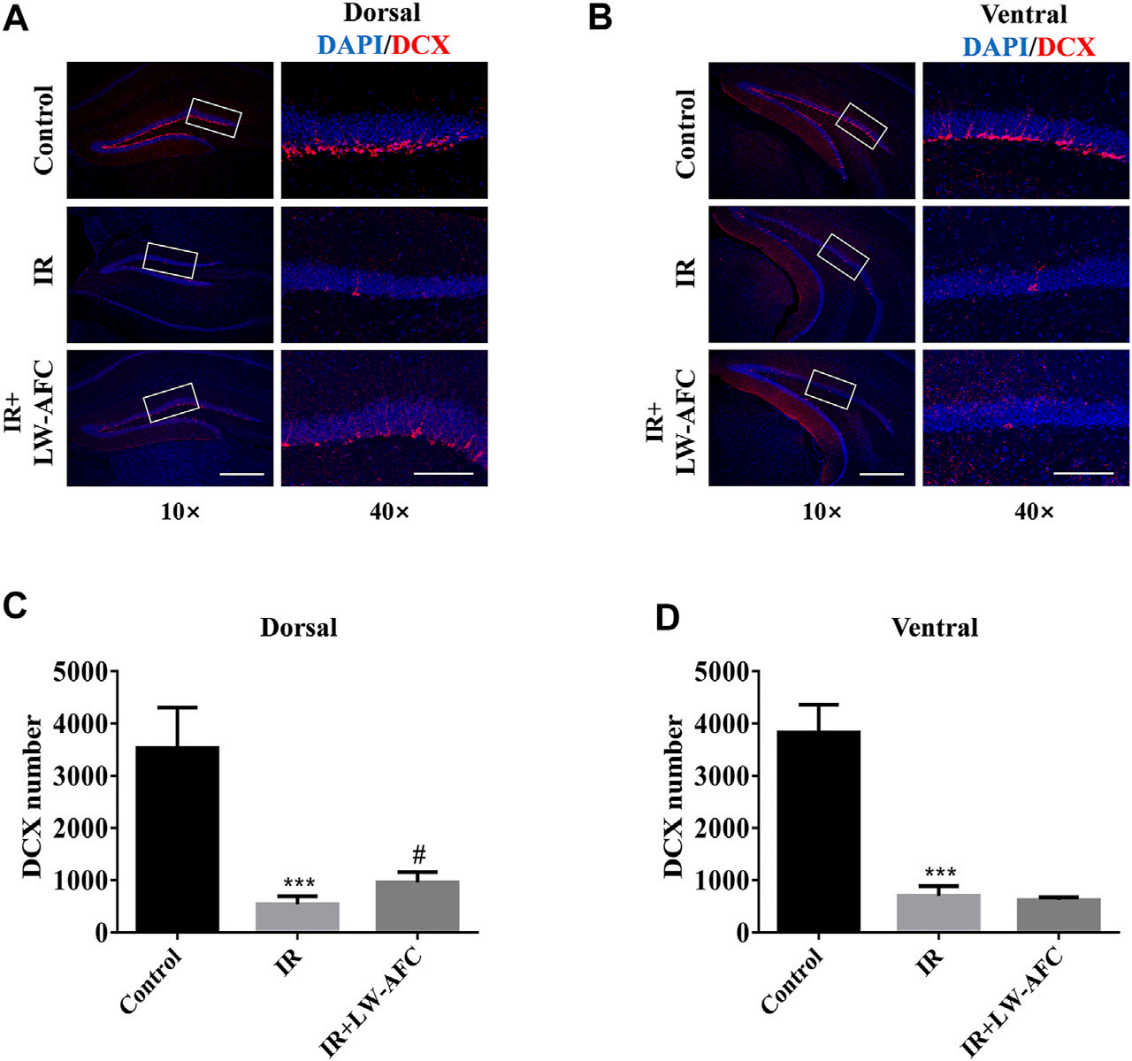
LW-AFC increases the number of immature neurons preferentially in the dorsal hippocampus of IR mice on day 1 after irradiation. Representative immunofluorescence images of DCX-positive cells in the **(A)** dorsal hippocampus and **(B)** ventral hippocampus. The area of white square frame in the left image indicates the enlarged image of DCX-positive cells in the right. The confocal images in the left are of 10×, and the enlarged images in the right are of 40×. Quantification of DCX-positive cells in the **(C)** dorsal hippocampus and the **(D)** ventral hippocampus. The scale bar in the left image = 300 μm, and the scale bar of the enlarged image = 100 μm. The values denote mean ± S.D., *n* = 4. ***p < 0.001, versus the control group; ^#^ *p <* 0.05, versus the IR group. Abbreviation: IR, irradiation.

To examine the effect of LW-AFC pretreatment on immature neurons in the dentate gyrus of the hippocampus along the dorsal–ventral axis in IR mice, the number of DCX-positive cells visualized by immunofluorescence staining was quantified. DCX is commonly used as a marker of immature neurons. On the first day after irradiation, compared with the control mice, the number of DCX-positive cells in the dorsal hippocampus of IR mice was significantly reduced and this change could be ameliorated significantly by LW-AFC pretreatment (*p* < 0.05) (Figures 5A,C). Compared with the control mice, the number of DCX-positive cells in the ventral hippocampus of IR mice was significantly decreased; however, this decrease could not be attenuated by the LW-AFC pretreatment (Figures 5B,D). The results showed that cranial irradiation reduced the number of immature neurons in both the dorsal and ventral hippocampus on the first day after irradiation. LW-AFC ameliorated cranial irradiation–induced reduction in the number of immature neurons in the dorsal hippocampus but had no effect on the ventral hippocampus.

Combined with the experimental data about the effect of LW-AFC on the proliferation of neural stem cells, we concluded that AHN was significantly damaged along the dorsal–ventral axis on the first day after irradiation and LW-AFC improved the impaired AHN predominantly in the dorsal hippocampus, which might be related to the improvement of behavioural dysfunctions.

### LW-AFC Pretreatment Ameliorated the Neurogenic Microenvironment in the Hippocampus of Irradiated Mice

The neurogenic microenvironment regulates the development and fate of neural stem cells, but cranial irradiation can lead to the alterations in the neurogenic microenvironment, which significantly contributes to the AHN impairment and cognitive and emotional dysfunctions from the cranial irradiation (Monje et al., 2002; Monje and Palmer, 2003; Jenrow et al., 2011; Ming and Song, 2011). Therefore, we further investigated whether LW-AFC pretreatment improved the neurogenic microenvironment in the hippocampus of IR mice on the first day after irradiation. We focused on the changes in the level of oxidative stress, neurotrophic and growth factors, and inflammation in the hippocampus of mice among three groups.

#### The Effect of LW-AFC Pretreatment on the Level of Oxidative Stress in the Hippocampus of Irradiated Mice

**FIGURE 6:**
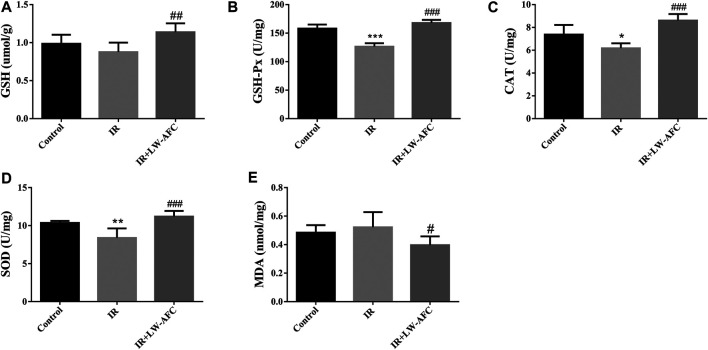
LW-AFC reduces the oxidative stress level in the hippocampus of IR mice on day 1 after irradiation. Concentrations of **(A)** GSH, **(B)** GSH-Px, **(C)** CAT, **(D)** SOD, and **(E)** MDA were detected. The values denote mean ± S.D., *n* = 6. *p < 0.05, **p < 0.01, and ***p < 0.001, versus the control group; ^#^ *p <* 0.05, ^##^ *p <* 0.01, and ^###^ *p <* 0.001, versus the IR group. Abbreviation: IR, irradiation.

To evaluate the effect of LW-AFC pretreatment on the level of oxidative stress in the hippocampus of IR mice, the contents of SOD, GSH-Px, GSH, CAT, and MDA were detected. The contents of SOD, GSH-Px, and CAT were reduced significantly, the GSH content tended to decrease, and the MDA content tended to increase in the hippocampus of IR mice, compared with the control mice. The LW-AFC pretreatment significantly increased the contents of SOD (*p* < 0.001), GSH-Px (*p* < 0.001), GSH (*p* < 0.01), and CAT (*p* < 0.001) and significantly reduced the content of MDA (*p* < 0.05) in the hippocampus of IR mice (Figure 6). The results showed that cranial irradiation increased the level of oxidative stress in the hippocampus of mice and this change could be reversed by LW-AFC.

#### The Effect of LW-AFC Pretreatment on the Level of Neurotrophic and Growth Factors in Hippocampus of Irradiated Mice

**FIGURE 7:**
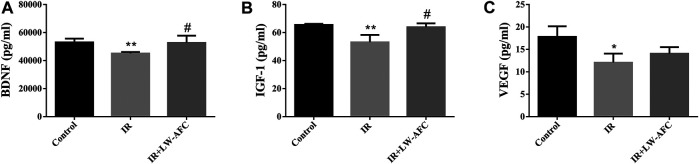
LW-AFC increases the level of neurotrophic and growth factors in the hippocampus of IR mice on day 1 after irradiation. Concentrations of **(A)** BDNF, **(B)** IGF-1, and **(C)** VEGF were detected. The values denote mean ± S.D., *n* = 4. *p < 0.05 and **p < 0.01, versus the control group. ^#^ *p <* 0.05, versus the IR group. Abbreviation: IR, irradiation.

To assess the effect of LW-AFC pretreatment on the level of neurotrophic and growth factors in the hippocampus of IR mice, the contents of BDNF, IGF-1, and VEGF were examined. The contents of BDNF, IGF-1, and VEGF were significantly decreased in the hippocampus of IR mice compared with the control mice. The contents of BDNF (*p* < 0.05) and IGF-1 (*p* < 0.05) were significantly increased in the hippocampus of IR mice by LW-AFC pretreatment (Figure 7). The results suggested that cranial irradiation reduced the level of neurotrophic and growth factors in the hippocampus of mice and this reduction could be mitigated by LW-AFC.

#### The Effect of LW-AFC Pretreatment on the Level of Inflammation in the Hippocampus of Irradiated Mice

**FIGURE 8:**
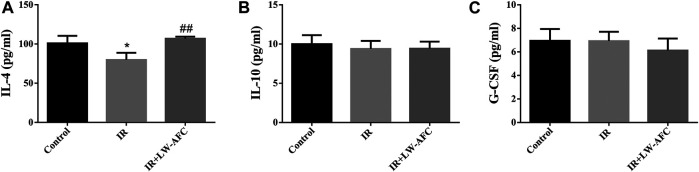
LW-AFC increases the level of anti-inflammatory factor in the hippocampus of IR mice on day 1 after irradiation. Concentrations of **(A)** IL-4, **(B)** IL-10, and **(C)** G-CSF were detected. The values denote mean ± S.D., *n* = 4. *p < 0.05, versus the control group; ^##^ *p <* 0.01, versus the IR group. Abbreviation: IR, irradiation.

To observe the effect of LW-AFC pretreatment on the level of inflammation in the hippocampus of IR mice, the proinflammatory factors and anti-inflammatory factors were detected. The proinflammatory factors included IL-1β, IL-6, IL-17A, and TNF-α. The anti-inflammatory factors included IL-4, IL-10, and G-CSF. The contents of IL-6, TNF-α, and IL-17A were significantly increased (Supplementary Figure S1) and the content of IL-4 was significantly decreased in the hippocampus of IR mice, compared with the control mice (Figure 8A). LW-AFC pretreatment significantly increased the content of IL-4 (*p* < 0.01) in the hippocampus of IR mice. The results demonstrated that cranial irradiation elevated the level of inflammation in the hippocampus of mice and this elevation could be attenuated by LW-AFC through modulating the anti-inflammatory factor.

Together, our study indicated that cranial irradiation disrupted the neurogenic microenvironment in the hippocampus of mice and LW-AFC ameliorated cranial irradiation–induced disturbance in the neurogenic microenvironment characterized by decrease in the level of oxidative stress and increase in the level of neurotrophic and growth factors as well as the anti-inflammatory factor, which were associated with LW-AFC–induced promotion of AHN and protection against cognitive and emotional dysfunctions.

## Discussion

Cranial radiotherapy is a necessary strategy in the treatment of primary and metastatic brain tumours, but it also leads to cognitive and emotional deficits, which are closely related to hippocampal dysfunctions. Some reports suggest that cranial irradiation–induced cognitive and emotional deficits are significantly linked to reduced AHN in the dentate gyrus of the hippocampus (Raber et al., 2004; Winocur et al., 2006). Moreover, cranial irradiation causes disturbance in the neurogenic microenvironment, which damages the neurogenic potential of the hippocampus and potentially contributes to these cognitive and emotional deficits (Monje et al., 2002; Rola et al., 2004). Promoting endogenous AHN and regulating the neurogenic microenvironment represent a new strategy for ameliorating cranial irradiation–induced functional deficits. In this study, we found that LW-AFC, when given 2 weeks before 7 Gy γ-ray cranial irradiation, could significantly improve cognitive and emotional deficits after cranial irradiation, and this effect was mediated by promoting AHN preferentially in the dorsal hippocampus and ameliorating the neurogenic microenvironment of the hippocampus in mice. It suggested that LW-AFC protected against the adverse effects and aided the recovery of hippocampal function following cranial irradiation.

Lots of studies have shown that the hippocampus is a structure with different anatomical connections, molecular features, and functions along the dorsal–ventral axis (Thierry et al., 2000; Kishi et al., 2006; Cenquizca and Swanson, 2007; Tanti et al., 2012). The dorsal hippocampus is mainly implicated in cognitive function, and the ventral hippocampus mainly mediates affective processes (Moser and Moser, 1998; Bannerman et al., 2004). AHN is a special neuroplasticity that produces the newborn neurons in the dentate gyrus of the hippocampus throughout the life of mammals (Gonçalves et al., 2016; Abbott and Nigussie, 2020). It is noted that AHN differs along the dorsal–ventral axis; for example, the dorsal hippocampus displays a faster maturation rate of newborn neurons than the ventral hippocampus (Snyder et al., 2009; Jinno, 2011; Snyder et al., 2012). AHN may also be preferentially affected in either the dorsal hippocampus or ventral hippocampus depending on the exact stimulation of the experiments, which suggests a regional specificity of AHN response; for example, a previous research revealed that environmental enrichment increased AHN in the dorsal hippocampus preferentially (Tanti et al., 2012). Therefore, it is reasonable to consider that the hippocampal function varies along the dorsal–ventral axis when AHN is examined, especially in experiments where drug therapy may affect AHN (O'Leary et al., 2012; Vivar et al., 2016; Zhou Q.-G. et al., 2016).

A previous study showed that the mice with ablation of AHN by cranial irradiation displayed more time in the open arm in the elevated plus maze and exhibited no difference in the immobility time in the forced swimming test, indicating reduced anxiety-like behaviour in neurogenesis-deficient mice (Tsai et al., 2015). Some studies revealed that cranial irradiation induced depression-like behaviour in mice (Wong-Goodrich et al., 2010; Son et al., 2014). Ablation of AHN by cranial irradiation had been previously reported to impair contextual fear conditioning in mice without affecting spatial memory, including the Morris water maze task and place recognition task of Y maze (Saxe et al., 2006). Another research showed that cranial irradiation reduced AHN and impaired performance of the object location task in rats; however, no overall cognitive impairment was observed (Lensu et al., 2020). Differences in studies about how AHN damage affected cognitive and emotional behaviour resulted from multiple factors, such as strain, gender, age of the animals, and experimental procedures.

Our study demonstrated that cranial irradiation decreased the number of proliferating neural stem cells and immature neurons in both the dorsal and ventral hippocampus on the first day after irradiation, suggesting that the AHN of mice was seriously damaged along the dorsal–ventral axis. Our results also revealed that mice with AHN damage exhibited the impairment of contextual fear memory, reduced anxiety level in the open field test, and showed an elevated tendency in the depression level in the tail suspension test on the first day after irradiation. Combined with the data on AHN and behaviour, we concluded that the impairment of AHN in both the dorsal and ventral hippocampus contributed to cognitive and emotional dysfunctions in mice.

There are currently no successful or effective interventions for cranial irradiation–induced cognitive and emotional dysfunctions (D’Antonio et al., 2014). Many TCM prescriptions “nourishing” in the TCM theory system have the effects of improving cognition and emotion and display the ability to promote AHN (Zhang et al., 2014; Sreenivasmurthy et al., 2017). LW, a classical TCM prescription, has been used clinically in the treatment of various diseases with signs of “kidney yin” deficiency (Hsieh et al., 2003; Zhou W. et al., 2016; Dai et al., 2016). A previous study showed that LW could promote AHN in adult rats (Lee et al., 2005). LW-AFC is an active fraction combination derived from LW. Previous studies had revealed that LW-AFC ameliorated cognitive and emotional dysfunctions in model mice of Alzheimer’s disease or stress (Zeng et al., 2019a; Huang et al., 2019; Cheng et al., 2020). In the present study, we further investigated the effects of LW-AFC on cognitive and emotional dysfunctions and AHN impairment along the dorsal–ventral axis induced by cranial irradiation. Our results showed that LW-AFC ameliorated behavioural dysfunctions in the contextual fear conditioning and open field tests on the first day after irradiation. In addition, in the tail suspension test, LW-AFC reduced the immobility time in IR mice. LW-AFC also improved cranial irradiation–induced impairment in the proliferation of neural stem cells and reduction in the number of immature neurons preferentially in the dorsal hippocampus on the first day after irradiation. Taken together, our study showed that LW-AFC ameliorated cranial irradiation–induced cognitive and emotional dysfunctions as well as promoted AHN preferentially in the dorsal hippocampus, and the amelioration of behavioural dysfunctions was related to the promotion of AHN.

Apart from the beneficial effect on AHN, we also investigated the effect of LW-AFC on the neurogenic microenvironment in the hippocampus of IR mice. The neurogenic microenvironment is composed of neural stem cells and their surrounding cells in the hippocampus, as well as the regulatory factors secreted by them (Zhao et al., 2008; Ming and Song, 2011; Nicola et al., 2015), and plays an important role in regulating the different stages of AHN (Mosher and Schaffer, 2018; Arredondo et al., 2020). Several reports indicated that regulating the changes in the neurogenic microenvironment might be an effective strategy for promoting AHN and improving cognitive and emotional dysfunctions following cranial irradiation, such as the reduction of inflammation and oxidative stress or the application of cytokines (Monje et al., 2003; Manda et al., 2009; Ramanan et al., 2009; Jenrow et al., 2010; Kim et al., 2010). Our research suggested that cranial irradiation induced disturbance in the neurogenic microenvironment of the hippocampus which was characterized by the increase in the level of oxidative stress and inflammation and the reduction in the level of neurotrophic and growth factors. It showed that LW-AFC increased the contents of SOD, GSH-Px, GSH, and CAT and decreased the content of MDA in the hippocampus of IR mice, as well as increased the contents of BDNF, IGF-1, and IL-4. The results demonstrated that LW-AFC ameliorated the disturbance in the neurogenic microenvironment in the hippocampus by reducing the level of oxidative stress and increasing the level of neurotrophic and growth factors as well as anti-inflammatory factor. Interestingly, LW-AFC promoted AHN and ameliorated cognitive and emotional dysfunctions despite having no effect on cranial irradiation–induced elevation in hippocampal proinflammatory cytokines, suggesting that the molecular signals triggered by LW-AFC could override this inhibitory effect.

In this study, there are some issues to be illustrated. First, AHN involves proliferation, differentiation, maturation, and migration with neural markers which can be used to quantify different stages (Faigle and Song, 2013). The proliferation and differentiation of neural stem cells have higher vulnerability to cranial irradiation (Bellinzona et al., 1996; Nagai et al., 2000; Lonergan et al., 2002; Andres-Mach et al., 2008). They are the two most important stages of AHN, which are closely linked to the generation of newborn neurons. They are positively correlated on the whole. Upon activation, the population of quiescent neural stem cells gives rise to a proliferative pool of neural stem cells and these cells continue to differentiate into immature neurons that express the marker of DCX in a multistep process (DeCarolis et al., 2013; Braun and Jessberger, 2014; Sibbe and Kulik, 2017). Second, there are currently no clinically available drugs for cranial irradiation treatment by promoting AHN. Although amifostine is the only proved radioprotective drug for radiotherapy in patients, its main pharmacological feature is antioxidant activity and it has many side effects (Anné, 2002; Vacha et al., 2003). We also searched the literature of similar studies and have found no suitable positive drug (Ji et al., 2014; Fan et al., 2017; Ji et al., 2020). Therefore, we also did not design positive drug treatment in our study. In addition, we have detected the neurogenic microenvironment in the whole hippocampus to observe the overall effect of LW-AFC. The change of the neurogenic microenvironment along the dorsal–ventral axis of the hippocampus is not examined. Further study is needed to fully solve this inadequacy.

## Conclusion

**FIGURE 9:**
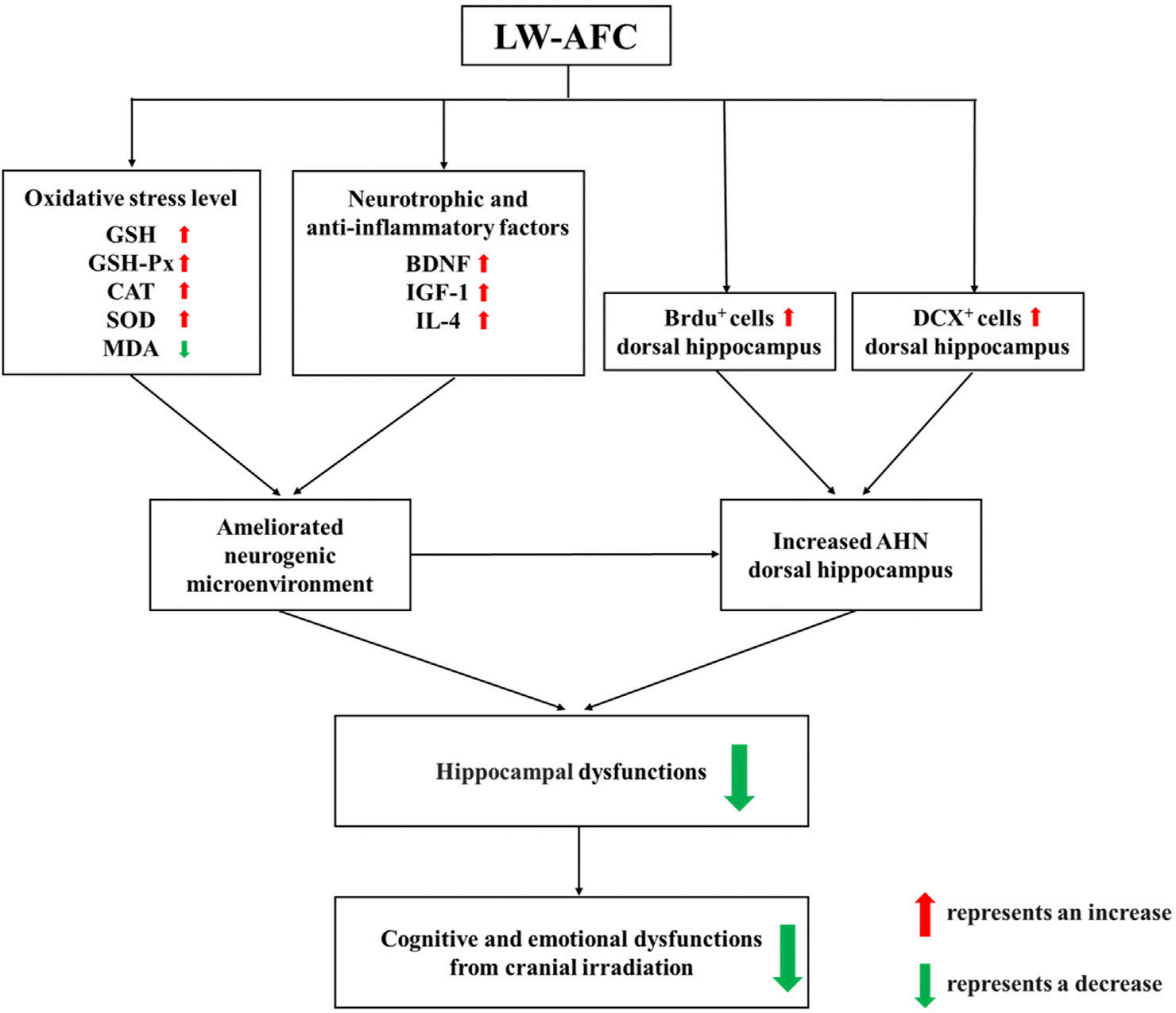
A schematic illustration of the protective mechanisms of LW-AFC against cranial irradiation–induced cognitive and emotional dysfunctions via increasing AHN in the dorsal hippocampus and modulating the neurogenic microenvironment.

In conclusion, first, our research demonstrated that cranial irradiation impaired AHN in both the dorsal and ventral hippocampus, which significantly contributed to the pathogenesis of cognitive and emotional dysfunctions following cranial irradiation. Second, LW-AFC significantly ameliorated cognitive and emotional dysfunctions after cranial irradiation. The underlying mechanisms included promoting AHN preferentially in the dorsal hippocampus and ameliorating disturbance in the neurogenic microenvironment. LW-AFC might be a promising therapeutic agent to treat cognitive and emotional dysfunctions induced by cranial irradiation (Figure 9), having the hope of safeguarding the health of brain tumour patients receiving cranial radiotherapy. Third, LW-AFC displayed a region-specific effect of neurogenic response along the dorsal–ventral axis of the hippocampus. We developed a scientific method to investigate whether TCM and its bioactive components could promote AHN accurately in different subregions of the hippocampus. Finally, the formula of TCM is advantageous in the treatment of complicated diseases for the integrative and synergistic effects exerted by its multiple components through multitargets. Our study provided a scientific method to develop the medication against cognitive and emotional dysfunctions induced by cranial irradiation from the resource of TCM.

## Data Availability

The original contributions presented in the study are included in the article/Supplementary Material; further inquiries can be directed to the corresponding authors.

## Abbreviations

AHN, adult hippocampal neurogenesis; BDNF, brain-derived neurotrophic factor; BrdU, bromodeoxyuridine; BSA, bovine serum albumin; CA-30, oligosaccharide fraction of LW-AFC; CAT, catalase; DCX, doublecortin; G-CSF, granulocyte colony–stimulating factor; GSH, glutathione; GSH-Px, glutathione peroxidase; HCl, hydrochloric acid; HPA, hypothalamic–pituitary–adrenal axis; HPLC, high-performance liquid chromatography; IGF-1, insulin-like growth factor-1; IL-1β, interleukin-1β; IR, irradiation; IR + LW-AFC, irradiation + LW-AFC; LW, Liuwei Dihuang decoction; LW-AFC, active fraction combination from Liuwei Dihuang decoction; LWB-B, polysaccharide fraction of LW-AFC; LWD-B, glycoside fraction of LW-AFC; MDA, malondialdehyde; PBS, phosphate-buffered saline; ROS, reactive oxygen species; SOD, superoxide dismutase; TCM, traditional Chinese medicine; TNF-α, tumour necrosis factor alpha; VEGF, vascular endothelial growth factor.

## References

[B1] Abbott L. C. Nigussie F. (2020). Adult Neurogenesis in the Mammalian Dentate Gyrus. Anat. Histol. Embryol. 49 (1), 3–16. 10.1111/ahe.12496 31568602

[B2] Acharya M. M. Christie L.-A. Lan M. L. Giedzinski E. Fike J. R. Rosi S. (2011). Human Neural Stem Cell Transplantation Ameliorates Radiation-Induced Cognitive Dysfunction. Cancer Res. 71 (14), 4834–4845. 10.1158/0008-5472.CAN-11-0027 21757460 PMC3517293

[B3] Andres-Mach M. Rola R. Fike J. R. (2008). Radiation Effects on Neural Precursor Cells in the Dentate Gyrus. Cell Tissue Res 331 (1), 251–262. 10.1007/s00441-007-0480-9 17786480

[B4] Anné P. R. (2002). Phase II Trial of Subcutaneous Amifostine in Patients Undergoing Radiation Therapy for Head and Neck Cancer. Semin. Oncol. 29 (6 Suppl. 19), 80–83. 10.1053/sonc.2002.37350b 12577250

[B5] Arredondo S. B. Valenzuela-Bezanilla D. Mardones M. D. Varela-Nallar L. (2020). Role of Wnt Signaling in Adult Hippocampal Neurogenesis in Health and Disease. Front. Cell Dev. Biol. 8, 860. 10.3389/fcell.2020.00860 33042988 PMC7525004

[B6] Balu D. T. Lucki I. (2009). Adult Hippocampal Neurogenesis: Regulation, Functional Implications, and Contribution to Disease Pathology. Neurosci. Biobehavioral Rev. 33 (3), 232–252. 10.1016/j.neubiorev.2008.08.007 PMC2671071 18786562

[B7] Bannerman D. M. Rawlins J. N. P. McHugh S. B. Deacon R. M. J. Yee B. K. Bast T. (2004). Regional Dissociations within the Hippocampus-Memory and Anxiety. Neurosci. Biobehavioral Rev. 28 (3), 273–283. 10.1016/j.neubiorev.2004.03.004 15225971

[B8] Bellinzona M. Gobbel G. T. Shinohara C. Fike J. R. (1996). Apoptosis Is Induced in the Subependyma of Young Adult Rats by Ionizing Irradiation. Neurosci. Lett. 208 (3), 163–166. 10.1016/0304-3940(96)12572-6 8733295

[B9] Braun S. M. G. Jessberger S. (2014). Review: Adult Neurogenesis and its Role in Neuropsychiatric Disease, Brain Repair and normal Brain Function. Neuropathol. Appl. Neurobiol. 40 (1), 3–12. 10.1111/nan.12107 24308291

[B10] Brenner D. J. Hall E. J. (2007). Computed Tomography - an Increasing Source of Radiation Exposure. N. Engl. J. Med. 357 (22), 2277–2284. 10.1056/NEJMra072149 18046031

[B11] Cenquizca L. A. Swanson L. W. (2007). Spatial Organization of Direct Hippocampal Field CA1 Axonal Projections to the Rest of the Cerebral Cortex. Brain Res. Rev. 56 (1), 1–26. 10.1016/j.brainresrev.2007.05.002 17559940 PMC2171036

[B12] Chen J. Teng D. Wu Z. Li W. Feng Y. Tang Y. (2021). Insights into the Molecular Mechanisms of Liuwei Dihuang Decoction via Network Pharmacology. Chem. Res. Toxicol. 34 (1), 91–102. 10.1021/acs.chemrestox.0c00359 33332098

[B13] Chen X. Wu H. Chen H. Wang Q. Xie X.-j. Shen J. (2019). Astragaloside VI Promotes Neural Stem Cell Proliferation and Enhances Neurological Function Recovery in Transient Cerebral Ischemic Injury via Activating EGFR/MAPK Signaling Cascades. Mol. Neurobiol. 56 (4), 3053–3067. 10.1007/s12035-018-1294-3 30088176

[B14] Cheng X.-R. Qi C.-H. Wang T.-X. Zhou W.-X. Zhang Y.-X. (2019). Characteristics of the Traditional Liu-Wei-Di-Huang Prescription Reassessed in Modern Pharmacology. Chin. J. Nat. Medicines 17 (2), 103–121. 10.1016/s1875-5364(19)30013-5 30797417

[B15] Cheng X. Huang Y. Zhang Y. Zhou W. (2020). LW-AFC, a New Formula from the Traditional Chinese Medicine Liuwei Dihuang Decoction, as a Promising Therapy for Alzheimer's Disease: Pharmacological Effects and Mechanisms. Adv. Pharmacol. 87, 159–177. 10.1016/bs.apha.2019.10.005 32089232

[B16] D'Antonio C. Passaro A. Gori B. Del Signore E. Migliorino M. R. Ricciardi S. (2014). Bone and Brain Metastasis in Lung Cancer: Recent Advances in Therapeutic Strategies. Ther. Adv. Med. Oncol. 6 (3), 101–114. 10.1177/1758834014521110 24790650 PMC3987652

[B17] Dai B. Wu Q. Zeng C. Zhang J. Cao L. Xiao Z. (2016). The Effect of Liuwei Dihuang Decoction on PI3K/Akt Signaling Pathway in Liver of Type 2 Diabetes Mellitus (T2DM) Rats with Insulin Resistance. J. Ethnopharmacology 192, 382–389. 10.1016/j.jep.2016.07.024 27401286

[B18] DeCarolis N. A. Mechanic M. Petrik D. Carlton A. Ables J. L. Malhotra S. (2013). In Vivo contribution of Nestin- and GLAST-Lineage Cells to Adult Hippocampal Neurogenesis. Hippocampus 23 (8), 708–719. 10.1002/hipo.22130 23554226 PMC3732558

[B19] Faigle R. Song H. (2013). Signaling Mechanisms Regulating Adult Neural Stem Cells and Neurogenesis. Biochim. Biophys. Acta (Bba) - Gen. Subjects 1830 (2), 2435–2448. 10.1016/j.bbagen.2012.09.002 PMC3541438 22982587

[B20] Fan X.-W. Liu H.-H. Wang H.-B. Chen F. Yang Y. Chen Y. (2017). Electroacupuncture Improves Cognitive Function and Hippocampal Neurogenesis after Brain Irradiation. Radiat. Res. 187 (6), 672–681. 10.1667/rr14561.1 28375680

[B21] Felice D. O'Leary O. F. Pizzo R. C. Cryan J. F. (2012). Blockade of the GABAB Receptor Increases Neurogenesis in the Ventral but Not Dorsal Adult hippocampus: Relevance to Antidepressant Action. Neuropharmacology 63 (8), 1380–1388. 10.1016/j.neuropharm.2012.06.066 22884610

[B22] Fike J. R. Rola R. Limoli C. L. (2007). Radiation Response of Neural Precursor Cells. Neurosurg. Clin. North America 18 (1), 115–127. 10.1016/j.nec.2006.10.010 17244559

[B23] Gonçalves J. T. Schafer S. T. Gage F. H. (2016). Adult Neurogenesis in the Hippocampus: From Stem Cells to Behavior. Cell 167 (4), 897–914. 10.1016/j.cell.2016.10.021 27814520

[B24] Hladik D. Tapio S. (2016). Effects of Ionizing Radiation on the Mammalian Brain. Mutat. Research/Reviews Mutat. Res. 770 (Pt B), 219–230. 10.1016/j.mrrev.2016.08.003 27919332

[B25] Hsieh M.-T. Cheng S.-J. Lin L.-W. Wang W.-H. Wu C.-R. (2003). The Ameliorating Effects of Acute and Chronic Administration of LiuWei Dihuang Wang on Learning Performance in Rodents. Biol. Pharm. Bull. 26 (2), 156–161. 10.1248/bpb.26.156 12576673

[B26] Hu Y. Liu X. Zhang T. Chen C. Dong X. Can Y. (2020). Behavioral and Biochemical Effects of KXS on Postmyocardial Infarction Depression. Front. Pharmacol. 11, 561817. 10.3389/fphar.2020.561817 32973539 PMC7481476

[B27] Huang T.-T. Zou Y. Corniola R. (2012a). Oxidative Stress and Adult Neurogenesis-Effects of Radiation and Superoxide Dismutase Deficiency. Semin. Cell Develop. Biol. 23 (7), 738–744. 10.1016/j.semcdb.2012.04.003 PMC3410958 22521481

[B28] Huang Y. Li D. Cheng B. Liu G. Zhang Y.-X. Zhou W.-X. (2019). Active Fraction Combination from Liuwei Dihuang Decoction (LW-AFC) Ameliorates Corticosterone-Induced Long-Term Potentiation (LTP) Impairment in Mice *In Vivo* . J. Ethnopharmacology 236, 147–154. 10.1016/j.jep.2019.03.002 30851370

[B29] Huang Y. Zhang H. Yang S. Qiao H. Zhou W. Zhang Y. (2012b). Liuwei Dihuang Decoction Facilitates the Induction of Long-Term Potentiation (LTP) in Senescence Accelerated Mouse/prone 8 (SAMP8) Hippocampal Slices by Inhibiting Voltage-dependent Calcium Channels (VDCCs) and Promoting N-Methyl-D-Aspartate Receptor (NMDA) Receptors. J. Ethnopharmacology 140 (2), 384–390. 10.1016/j.jep.2012.01.030 22310556

[B30] Imayoshi I. Sakamoto M. Ohtsuka T. Takao K. Miyakawa T. Yamaguchi M. (2008). Roles of Continuous Neurogenesis in the Structural and Functional Integrity of the Adult Forebrain. Nat. Neurosci. 11 (10), 1153–1161. 10.1038/nn.2185 18758458

[B31] Itoh T. Murai S. Saito H. Masuda Y. (1998). Effects of Single and Repeated Administrations of Toki-Shakuyaku-San on the Concentrations of Brain Neurotransmitters in Mice. Methods Find Exp. Clin. Pharmacol. 20 (1), 11–17. 10.1358/mf.1998.20.1.485617 9575477

[B32] Jenrow K. A. Brown S. L. Liu J. Kolozsvary A. Lapanowski K. Kim J. H. (2010). Ramipril Mitigates Radiation-Induced Impairment of Neurogenesis in the Rat Dentate Gyrus. Radiat. Oncol. 5, 6. 10.1186/1748-717x-5-6 20122169 PMC2825515

[B33] Jenrow K. A. Liu J. Brown S. L. Kolozsvary A. Lapanowski K. Kim J. H. (2011). Combined Atorvastatin and Ramipril Mitigate Radiation-Induced Impairment of Dentate Gyrus Neurogenesis. J. Neurooncol. 101 (3), 449–456. 10.1007/s11060-010-0282-x 20617366

[B34] Ji J.-f. Ji S.-j. Sun R. Li K. Zhang Y. Zhang L.-y. (2014). Forced Running Exercise Attenuates Hippocampal Neurogenesis Impairment and the Neurocognitive Deficits Induced by Whole-Brain Irradiation via the BDNF-Mediated Pathway. Biochem. Biophysical Res. Commun. 443 (2), 646–651. 10.1016/j.bbrc.2013.12.031 24333433

[B35] Ji S. Wu H. Ding X. Chen Q. Jin X. Yu J. (2020). Increased Hippocampal TrkA Expression Ameliorates Cranial Radiation-induced Neurogenesis Impairment and Cognitive Deficit via PI3K/AKT Signaling. Oncol. Rep. 44 (6), 2527–2536. 10.3892/or.2020.7782 33125501 PMC7640353

[B36] Jinno S. (2011). Topographic Differences in Adult Neurogenesis in the Mouse hippocampus: a Stereology-Based Study Using Endogenous Markers. Hippocampus 21 (5), 467–480. 10.1002/hipo.20762 20087889

[B37] Kim J.-S. Yang M. Jang H. Oui H. Kim S.-H. Shin T. (2010). Granulocyte-colony Stimulating Factor Ameliorates Irradiation-Induced Suppression of Hippocampal Neurogenesis in Adult Mice. Neurosci. Lett. 486 (1), 43–46. 10.1016/j.neulet.2010.09.041 20854880

[B38] Kishi T. Tsumori T. Yokota S. Yasui Y. (2006). Topographical Projection from the Hippocampal Formation to the Amygdala: a Combined Anterograde and Retrograde Tracing Study in the Rat. J. Comp. Neurol. 496 (3), 349–368. 10.1002/cne.20919 16566004

[B39] Lee K.-S. Lim B.-V. Chang H.-K. Yang H.-Y. Bahn G.-H. Paik E.-K. (2005). Liuweidihuang-tang Improves Spatial Memory Function and Increases Neurogenesis in the Dentate Gyrus in Rats. Fitoterapia 76 (6), 514–519. 10.1016/j.fitote.2005.04.022 15972247

[B40] Lensu S. Waselius T. Mäkinen E. Kettunen H. Virtanen A. Tiirola M. (2021). Irradiation of the Head Reduces Adult Hippocampal Neurogenesis and Impairs Spatial Memory, but Leaves Overall Health Intact in Rats. Eur. J. Neurosci. 53, 1885–1904. 10.1111/ejn.15102 33382141

[B41] Limoli C. L. Giedzinski E. Baure J. Rola R. Fike J. R. (2007). Redox Changes Induced in Hippocampal Precursor Cells by Heavy Ion Irradiation. Radiat. Environ. Biophys. 46 (2), 167–172. 10.1007/s00411-006-0077-9 17103219

[B42] Lin Y.-C. Wu C.-R. Lin C.-J. Hsieh M.-T. (2003). The Ameliorating Effects of Cognition-Enhancing Chinese Herbs on Scopolamine-And MK-801-Induced Amnesia in Rats. Am. J. Chin. Med. 31 (4), 543–549. 10.1142/s0192415x03001302 14587877

[B43] Lonergan P. E. Martin D. S. D. Horrobin D. F. Lynch M. A. (2002). Neuroprotective Effect of Eicosapentaenoic Acid in Hippocampus of Rats Exposed to γ-Irradiation. J. Biol. Chem. 277 (23), 20804–20811. 10.1074/jbc.M202387200 11912218

[B44] Manda K. Reiter R. J. (2010). Melatonin Maintains Adult Hippocampal Neurogenesis and Cognitive Functions after Irradiation. Prog. Neurobiol. 90 (1), 60–68. 10.1016/j.pneurobio.2009.10.019 19857546

[B45] Manda K. Ueno M. Anzai K. (2009). Cranial Irradiation-Induced Inhibition of Neurogenesis in Hippocampal Dentate Gyrus of Adult Mice: Attenuation by Melatonin Pretreatment. J. Pineal Res. 46 (1), 71–78. 10.1111/j.1600-079X.2008.00632.x 18798786

[B46] Martel G. Uchida S. Hevi C. Chévere-Torres I. Fuentes I. Park Y. J. (2016). Genetic Demonstration of a Role for Stathmin in Adult Hippocampal Neurogenesis, Spinogenesis, and NMDA Receptor-dependent Memory. J. Neurosci. 36 (4), 1185–1202. 10.1523/jneurosci.4541-14.2016 26818507 PMC4728724

[B47] Ming G.-l. Song H. (2011). Adult Neurogenesis in the Mammalian Brain: Significant Answers and Significant Questions. Neuron 70 (4), 687–702. 10.1016/j.neuron.2011.05.001 21609825 PMC3106107

[B48] Ming G.-l. Song H. (2005). Adult Neurogenesis in the Mammalian central Nervous System. Annu. Rev. Neurosci. 28, 223–250. 10.1146/annurev.neuro.28.051804.101459 16022595

[B49] Mizumatsu S. Monje M. L. Morhardt D. R. Rola R. Palmer T. D. Fike J. R. (2003). Extreme Sensitivity of Adult Neurogenesis to Low Doses of X-Irradiation. Cancer Res. 63 (14), 4021–4027. 12874001

[B50] Monje M. (2008). Cranial Radiation Therapy and Damage to Hippocampal Neurogenesis. Dev. Disabil. Res. Revs 14 (3), 238–242. 10.1002/ddrr.26 18924155

[B51] Monje M. L. Mizumatsu S. Fike J. R. Palmer T. D. (2002). Irradiation Induces Neural Precursor-Cell Dysfunction. Nat. Med. 8 (9), 955–962. 10.1038/nm749 12161748

[B52] Monje M. L. Palmer T. (2003). Radiation Injury and Neurogenesis. Curr. Opin. Neurol. 16 (2), 129–134. 10.1097/01.wco.0000063772.81810.b7 12644738

[B53] Monje M. L. Toda H. Palmer T. D. (2003). Inflammatory Blockade Restores Adult Hippocampal Neurogenesis. Science 302 (5651), 1760–1765. 10.1126/science.1088417 14615545

[B54] Moser M.-B. Moser E. I. (1998). Functional Differentiation in the hippocampus. Hippocampus 8 (6), 608–619. 10.1002/(sici)1098-1063(1998)8:6<608::aid-hipo3>3.0.co;2-7 9882018

[B55] Mosher K. I. Schaffer D. V. (2018). Influence of Hippocampal Niche Signals on Neural Stem Cell Functions during Aging. Cell Tissue Res 371 (1), 115–124. 10.1007/s00441-017-2709-6 29124394 PMC5750097

[B56] Nagai R. Tsunoda S. Hori Y. Asada H. (2000). Selective Vulnerability to Radiation in the Hippocampal Dentate Granule Cells. Surg. Neurol. 53 (5), 503–507. 10.1016/s0090-3019(00)00214-7 10874152

[B57] Naylor A. S. Bull C. Nilsson M. K. L. Zhu C. Bjork-Eriksson T. Eriksson P. S. (2008). Voluntary Running Rescues Adult Hippocampal Neurogenesis after Irradiation of the Young Mouse Brain. Proc. Natl. Acad. Sci. 105 (38), 14632–14637. 10.1073/pnas.0711128105 18765809 PMC2567198

[B58] Nicola Z. Fabel K. Kempermann G. (2015). Development of the Adult Neurogenic Niche in the hippocampus of Mice. Front. Neuroanat. 9, 53. 10.3389/fnana.2015.00053 25999820 PMC4423450

[B59] Nishiyama N. Zhou Y. Takashina K. Saito H. (1994). Effects of DX-9386, a Traditional Chinese Preparation, on Passive and Active Avoidance Performances in Mice. Biol. Pharm. Bull. 17 (11), 1472–1476. 10.1248/bpb.17.1472 7703966

[B60] O'Leary O. F. O'Connor R. M. Cryan J. F. (2012). Lithium-induced Effects on Adult Hippocampal Neurogenesis Are Topographically Segregated along the Dorso-Ventral axis of Stressed Mice. Neuropharmacology 62 (1), 247–255. 10.1016/j.neuropharm.2011.07.015 21803056

[B61] Raber J. Rola R. LeFevour A. Morhardt D. Curley J. Mizumatsu S. (2004). Radiation-induced Cognitive Impairments Are Associated with Changes in Indicators of Hippocampal Neurogenesis. Radiat. Res. 162 (1), 39–47. 10.1667/rr3206 15222778

[B62] Ramanan S. Kooshki M. Zhao W. Hsu F.-C. Riddle D. R. Robbins M. E. (2009). The PPARα Agonist Fenofibrate Preserves Hippocampal Neurogenesis and Inhibits Microglial Activation after Whole-Brain Irradiation. Int. J. Radiat. Oncology*Biology*Physics 75 (3), 870–877. 10.1016/j.ijrobp.2009.06.059 PMC2789462 19801103

[B63] Rola R. Otsuka S. Obenaus A. Nelson G. A. Limoli C. L. VandenBerg S. R. (2004). Indicators of Hippocampal Neurogenesis Are Altered by56Fe-Particle Irradiation in a Dose-dependent Manner. Radiat. Res. 162 (4), 442–446. 10.1667/rr3234 15447038

[B64] Saxe M. D. Battaglia F. Wang J.-W. Malleret G. David D. J. Monckton J. E. (2006). Ablation of Hippocampal Neurogenesis Impairs Contextual Fear Conditioning and Synaptic Plasticity in the Dentate Gyrus. Proc. Natl. Acad. Sci. 103 (46), 17501–17506. 10.1073/pnas.0607207103 17088541 PMC1859958

[B65] Shang B. Zhang H. Lu Y. Zhou X. Wang Y. Ma M. (2020). Insights from the Perspective of Traditional Chinese Medicine to Elucidate Association of Lily Disease and Yin Deficiency and Internal Heat of Depression. Evidence-Based Complement. Altern. Med. 2020, 1–8. 10.1155/2020/8899079 PMC7710406 33299463

[B66] Shen W. Sun X. Zhu M. Wang Y. Jiang N. Zhou W. (2018). Effects and Mechanisms of Liuwei Dihuang Active Fraction Combination on Anxiety-And Depression-like Behaviour Induced by Sleep Deprivation in Mice. Int. J. Pharm. Res. 45 (12), 920–927.

[B67] Sibbe M. Kulik A. (2017). GABAergic Regulation of Adult Hippocampal Neurogenesis. Mol. Neurobiol. 54 (7), 5497–5510. 10.1007/s12035-016-0072-3 27599499

[B68] Snyder J. S. Ferrante S. C. Cameron H. A. (2012). Late Maturation of Adult-Born Neurons in the Temporal Dentate Gyrus. PLoS One 7 (11), e48757. 10.1371/journal.pone.0048757 23144957 PMC3492442

[B69] Snyder J. S. Radik R. Wojtowicz J. M. Cameron H. A. (2009). Anatomical Gradients of Adult Neurogenesis and Activity: Young Neurons in the Ventral Dentate Gyrus Are Activated by Water Maze Training. Hippocampus 19 (4), 360–370. 10.1002/hipo.20525 19004012 PMC2798730

[B70] Son Y. Yang M. Kim J.-S. Kim J. Kim S.-H. Kim J.-C. (2014). Hippocampal Dysfunction during the Chronic Phase Following a Single Exposure to Cranial Irradiation. Exp. Neurol. 254, 134–144. 10.1016/j.expneurol.2014.01.018 24491956

[B71] Son Y. Yang M. Wang H. Moon C. (2015). Hippocampal Dysfunctions Caused by Cranial Irradiation: a Review of the Experimental Evidence. Brain Behav. Immun. 45, 287–296. 10.1016/j.bbi.2015.01.007 25596174

[B72] Sreenivasmurthy S. Liu J.-Y. Song J.-X. Yang C.-B. Malampati S. Wang Z.-Y. (2017). Neurogenic Traditional Chinese Medicine as a Promising Strategy for the Treatment of Alzheimer's Disease. Ijms 18 (2), 272. 10.3390/ijms18020272 PMC5343808 28134846

[B73] Tada E. Parent J. M. Lowenstein D. H. Fike J. R. (2000). X-irradiation Causes a Prolonged Reduction in Cell Proliferation in the Dentate Gyrus of Adult Rats. Neuroscience 99 (1), 33–41. 10.1016/s0306-4522(00)00151-2 10924950

[B74] Tanti A. Rainer Q. Minier F. Surget A. Belzung C. (2012). Differential Environmental Regulation of Neurogenesis along the Septo-Temporal axis of the hippocampus. Neuropharmacology 63 (3), 374–384. 10.1016/j.neuropharm.2012.04.022 22561281

[B75] Thierry A.-M. Gioanni Y. Dégénétais E. Glowinski J. (2000). Hippocampo-prefrontal Cortex Pathway: Anatomical and Electrophysiological Characteristics. Hippocampus 10 (4), 411–419. 10.1002/1098-1063(2000)10:4<411::aid-hipo7>3.0.co;2-a 10985280

[B76] Tsai C.-Y. Tsai C.-Y. Arnold S. J. Huang G.-J. (2015). Ablation of Hippocampal Neurogenesis in Mice Impairs the Response to Stress during the Dark Cycle. Nat. Commun. 6, 8373. 10.1038/ncomms9373 26415720 PMC4598562

[B77] Vacha P. Fehlauer F. Mahlmann B. Marx M. Hinke A. Sommer K. (2003). Randomized Phase III Trial of Postoperative Radiochemotherapy ± Amifostine in Head and Neck Cancer. Strahlenther Onkol 179 (6), 385–389. 10.1007/s00066-003-1016-1 12789464

[B78] Vivar C. Peterson B. D. van Praag H. (2016). Running Rewires the Neuronal Network of Adult-Born Dentate Granule Cells. Neuroimage 131, 29–41. 10.1016/j.neuroimage.2015.11.031 26589333 PMC6003242

[B79] Wang J.-H. Lei X. Cheng X.-R. Zhang X.-R. Liu G. Cheng J.-P. (2016). LW-AFC, a New Formula Derived from Liuwei Dihuang Decoction, Ameliorates Behavioral and Pathological Deterioration via Modulating the Neuroendocrine-Immune System in PrP-hAβPPswe/PS1ΔE9 Transgenic Mice. Alz Res. Ther. 8 (1), 57. 10.1186/s13195-016-0226-6 PMC5154149 27964740

[B80] Wang J. Cheng X. Zeng J. Yuan J. Wang Z. Zhou W. (2017a). LW-AFC Effects on N-Glycan Profile in Senescence-Accelerated Mouse Prone 8 Strain, a Mouse Model of Alzheimer's Disease. Aging Dis. 8 (1), 101–114. 10.14336/AD.2016.0522 28203484 PMC5287383

[B81] Wang J. Zhang X. Cheng X. Cheng J. Liu F. Xu Y. (2017b). LW-AFC, A New Formula Derived from Liuwei Dihuang Decoction, Ameliorates Cognitive Deterioration and Modulates Neuroendocrine-Immune System in SAMP8 Mouse. Car 14 (2), 221–238. 10.2174/1567205013666160603001637 27335033

[B82] Winocur G. Wojtowicz J. M. Sekeres M. Snyder J. S. Wang S. (2006). Inhibition of Neurogenesis Interferes with Hippocampus-dependent Memory Function. Hippocampus 16 (3), 296–304. 10.1002/hipo.20163 16411241

[B83] Wong-Goodrich S. J. E. Pfau M. L. Flores C. T. Fraser J. A. Williams C. L. Jones L. W. (2010). Voluntary Running Prevents Progressive Memory Decline and Increases Adult Hippocampal Neurogenesis and Growth Factor Expression after Whole-Brain Irradiation. Cancer Res. 70 (22), 9329–9338. 10.1158/0008-5472.CAN-10-1854 20884629 PMC2982943

[B84] Yang S. Zhou W. Zhang Y. Yan C. Zhao Y. (2006). Effects of Liuwei Dihuang Decoction on Ion Channels and Synaptic Transmission in Cultured Hippocampal Neuron of Rat. J. Ethnopharmacology 106 (2), 166–172. 10.1016/j.jep.2005.12.017 16442252

[B85] Zeng J. Cheng B. Huang Y. Zhang X. Wang C. Sun N. (2019b). Active Fraction Combination from Liuwei Dihuang Decoction (LW-AFC) Alleviated the LPS-Induced Long-Term Potentiation Impairment and Glial Cells Activation in Hippocampus of Mice by Modulating Immune Responses. Evidence-Based Complement. Altern. Med. 2019, 1–13. 10.1155/2019/3040972 PMC6766147 31636681

[B86] Zeng J. Cheng X. Zhou W. Zhang Y. (2019a). Effects of LW-AFC on Anxiety-like Behaviour and Immune Function in Corticosterone-Induced Mice. Chin. J. Pharmacol. Toxicol. 33 (09), 48–49.

[B87] Zhang E. Shen J. So K. F. (2014). Chinese Traditional Medicine and Adult Neurogenesis in the hippocampus. J. Traditional Complement. Med. 4 (2), 77–81. 10.4103/2225-4110.130372 PMC4003705 24860729

[B88] Zhang W. Zhao R. Li X. Cui X. Zhao Z. Mao Y. (2016). Effect of Yi-Nao-Jie-Yu Decoction on γ-aminobutyric Acid Type A Receptor in the hippocampus and Serum Inflammatory Factors in a Rat Model of Poststroke Anxiety. Ndt 12, 2827–2837. 10.2147/ndt.s115116 PMC5098770 27843317

[B89] Zhao C. Deng W. Gage F. H. (2008). Mechanisms and Functional Implications of Adult Neurogenesis. Cell 132 (4), 645–660. 10.1016/j.cell.2008.01.033 18295581

[B90] Zheng J. Jiang Y.-Y. Xu L.-C. Ma L.-Y. Liu F.-Y. Cui S. (2017). Adult Hippocampal Neurogenesis along the Dorsoventral Axis Contributes Differentially to Environmental Enrichment Combined with Voluntary Exercise in Alleviating Chronic Inflammatory Pain in Mice. J. Neurosci. 37 (15), 4145–4157. 10.1523/jneurosci.3333-16.2017 28292830 PMC6596585

[B91] Zhou Q.-G. Lee D. Ro E. J. Suh H. (2016a). Regional-specific Effect of Fluoxetine on Rapidly Dividing Progenitors along the Dorsoventral axis of the hippocampus. Sci. Rep. 6, 35572. 10.1038/srep35572 27759049 PMC5069667

[B92] Zhou W. Cheng X. Zhang Y. (2016b). Effect of Liuwei Dihuang Decoction, a Traditional Chinese Medicinal Prescription, on the Neuroendocrine Immunomodulation Network. Pharmacol. Ther. 162, 170–178. 10.1016/j.pharmthera.2016.02.004 26896567

[B93] Zhu M. Zhou W. Jiang N. (2018). Effect and Mechanism of LW-AFC on Chronic Unpredictable Mild Stress-Induced Mood and Cognition Impairment of Mice. Chin. J. Pharmacol. Toxicol. 32 (04), 344–345.

