# Supplementary material for: Active Fraction Combination From Liuwei Dihuang Decoction Improves Adult Hippocampal Neurogenesis and Neurogenic Microenvironment in Cranially Irradiated Mice
Source: Front Pharmacol. 2021 Sep 23;12:717719. doi: 10.3389/fphar.2021.717719 (PMC8495126; doi:10.3389/fphar.2021.717719)
Supplement: Supplementary file 1 [file DataSheet1.docx]

**Supplementary Material. The methods of the preparation of LW-AFC**

LW-AFC was prepared from LW as follows. (1) Six herbs of LW, including Rehmannia glutinosa (Gaertn.) DC. [Orobanchaceae; Rehmanniae radix], Dioscorea oppositifolia L. [Dioscoreaceae; Dioscoreae rhizoma], Cornus officinalis Siebold & Zucc. [Cornaceae; Corni fructus], Alisma plantago-aquatica L. [Alismataceae; Alismatis rhizoma], Poria cocos (Schw.) Wolf [Polyporaceae; Poria], and Paeonia × suffruticosa Andrews [Paeoniaceae; Moutan cortex], were mixed according to the dry weight ratio of 8:4:4:3:3:3. The mixture of herb materials were decocted within 10 volume of deionized water with boiling refluxing thrice, 2h per time. After finishing the extraction, the materials were 6-layer gauze filtered to yield three extraction solutions at 50°C, allowed them to room temperature, centrifuged (2500rpm/min, 25min). The supernatants were combined and then concentrated under reduced pressure (relative density was 1.09 at 20°C) into quintessence.

(2) The quintessence was left in 30% ethanol overnight at room temperature. After centrifugation (2500rpm/min, 25min) and washing sedimentation with 30% ethanol three times, the sedimentation was decanted. All the supernatant was collected and combined, then concentrated under reduced pressure (relative density was 1.17 at 20°C).

(3) The concentrated extract was left in 60% ethanol overnight at room temperature, then centrifuged (2500rpm/min, 25min), the supernatant (LWD) was collected, the sedimentation left in deionized water and concentrated under reduced pressure (relative density was 1.09 at 20°C) three times, then dried at 70°C to obtain polysaccharide fraction (LWB-B).

(4) The supernatant (LWD) was left in 60% ethanol and concentrated to 1/10 original volume under reduced pressure, then added 20% of its original volume of deionized water, concentrated three times (relative density was 1.285 at 20°C), then dissolved in a sufficient quantity of deionized water and eluted in turn by deionized water (6 column volume) and 30% ethanol (4 column volume) on macroporous adsorptive resins (DIAION HP20, Φ150×1500mm, diameter height ratio was 1/9) (Mitsubishi Chemical Corporation, Minato-ku, Tokyo, Japan) with 19.1 cm/h sample flow rate and 19.1 cm/h eluent flow rate. 30% ethanol elution of LWD was concentrated (relative density was 1.05 at 20°C), cryodesiccated to obtain glycosides fraction (LWD-B).

(5) Water elution of LWD was concentrated (relative density was 1.192 at 20°C), eluted in turn by 5% ethanol (6 column volume) and 30% ethanol (2 column volume) on active carbon absorption column (GH-15,Φ150×1500mm, diameter height ratio was 1/9) (Guanghuajingke Activate Carbon Ltd., Beijing, China) with 19.1 cm/h sample flow rate and 25.0 cm/h eluent flow rate. 5% ethanol elution was decanted. 30% ethanol elution was concentrated to 1/20 of its original volume under reduced pressure, then added 20% of its original volume of deionized water, concentrated three times (relative density was 1.10–1.15 at 20°C), cryodesiccated to obtain oligosaccharide fraction (CA-30).

(6) LW-AFC was composed of 20.3% polysaccharide fraction (LWB-B), 15.1% glycosides fraction (LWD-B) and 64.6% oligosaccharide fraction (CA-30) in the dry weight ratio.
