# Supplementary material for: Active Fraction Combination From Liuwei Dihuang Decoction Improves Adult Hippocampal Neurogenesis and Neurogenic Microenvironment in Cranially Irradiated Mice
Source: Front Pharmacol. 2021 Sep 23;12:717719. doi: 10.3389/fphar.2021.717719 (PMC8495126; doi:10.3389/fphar.2021.717719)
Supplement: Supplementary file 2 [file Image1.pdf]

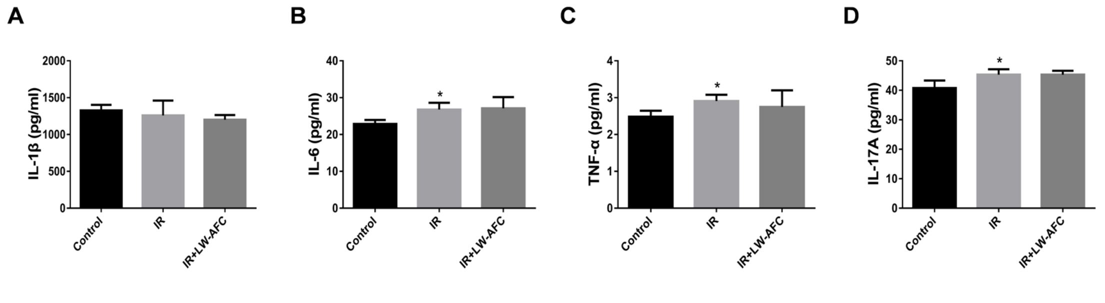

Figure S1. Effect of LW-AFC on pro-inflammatory factors in hippocampus of IR mice on day 1 after irradiation. Concentration of **(A)** (IL-1β), **(B)** (IL-6), **(C)** (TNF-α), and **(D)** (IL-17A) were detected. The values are mean ± S.D., n=4. *P＜0.05, versus the control group. IR, irradiation.
